# Male reproductive health under environmental, pharmacological, and disease influences: a scoping review

**DOI:** 10.3389/fendo.2026.1922443

**Published:** 2026-10-02

**Authors:** Sylvester Chibueze Izah, Kingsley Excel Dunga, Ebinyo Rebecca Aseibai, Nsikak Godwin Etim, Matthew Chidozie Ogwu, Vivian Ibienebakabobo Promise, Esther Ugo Alum, Irene Nneka Ohanusi

**Affiliations:** 1 Department of Community Medicine, Faculty of Clinical Sciences, Bayelsa Medical University, Yenagoa, Bayelsa State, Nigeria; 2 Department of Microbiology, Faculty of Science, Bayelsa Medical University, Yenagoa, Bayelsa State, Nigeria; 3 Department of Medical Laboratory Science, Kingsley Ozumba Mbadiwe University, Ogboko, Imo State, Nigeria; 4 Department of Microbiology, Federal University Otuoke, Bayelsa State, Nigeria; 5 Niger-Delta Institute for Emerging and Re-emerging Infectious Diseases, Federal University Otuoke, Yenagoa, Bayelsa State, Nigeria; 6 Goodnight Family Department of Sustainable Development, Appalachian State University, 212 Living Learning Center, Boone, NC, United States; 7 Department of Public Health, Faculty of Health Sciences, Bayelsa Medical University, Bayelsa State, Nigeria; 8 Department of Research and Publications, Kampala International University, Kampala, Uganda; 9 Department of Microbiology, Faculty of Natural Sciences, Hezekiah University Umudi, Nkwerre, Imo State, Nigeria

**Keywords:** environmental toxicants, fertility preservation, male infertility, male reproductive health, mixed exposures, pharmaceuticals, semen quality

## Abstract

Male reproductive health reflects coordinated spermatogenesis, endocrine regulation, reproductive-tract patency, sexual function, and sperm genomic integrity. This scoping review maps evidence on environmental exposures, pharmacological agents and substance use, and acute or chronic diseases that may influence these domains, with emphasis on biological mechanisms, clinical assessment, prevention, and public-health implications. PubMed was the primary bibliographic database; Google Scholar and official grey literature/guidelines were supplementary sources. The evidence map comprised 105 unique sources: 99 PubMed-indexed peer-reviewed sources and six official grey-literature/guideline sources. Google Scholar supported supplementary discovery and citation chaining, and overlapping PubMed records were counted once after de-duplication; no unique Google-Scholar-only journal source remained in the mapped set. Four additional references were used exclusively to guide scoping-review methodology and reporting, giving 109 references in the complete bibliography. Evidence was charted by exposure or condition, study design, reproductive outcome, quantitative finding where available, mechanistic interpretation, and clinical implication. The mapped literature identifies endocrine-disrupting chemicals, pesticides, metals, air pollution, occupational heat, selected medicines and gonadotoxic treatments, metabolic and infectious diseases, varicocele, genetic disorders, and systemic illness as relevant reproductive-health contexts, but the strength and causal interpretability of evidence vary substantially. Recurrent mechanisms include oxidative stress, inflammation, endocrine disturbance, mitochondrial dysfunction, vascular impairment, sperm DNA damage, and epigenetic alteration. The review supports couple-centred evaluation, source control of avoidable exposures, treatment of reversible disease, fertility preservation before gonadotoxic therapy, and specialist-led medication decisions.

## 1 Introduction

Male reproductive health is broader than the capacity to father a child. It includes testicular development and function, androgen production, sperm formation and transport, erection and ejaculation, and informed reproductive decision-making (1, 2). A man may have normal sperm production but erectile or ejaculatory dysfunction; another may have no sexual symptoms but azoospermia, sperm DNA damage, or obstruction (3, 4). Treating male reproductive health as only a semen-count issue can therefore miss endocrine disease, cardiovascular illness, mental-health needs, relationship concerns, occupational hazards, and preventable toxic exposures (2, 5).

Normal reproductive capacity rests on several linked systems. The hypothalamic-pituitary-gonadal axis regulates steroidogenesis and spermatogenesis, while the testes and epididymides produce and mature sperm (6, 7). Accessory glands shape seminal fluid, and intact vascular and neural pathways support erection and ejaculation (8; 9). Environmental toxicants, illness, heat, inflammation, medicines, nutrition, and psychosocial stress can disturb more than one system simultaneously (10, 11).

These systems could also explain why a normal result in one domain cannot rule out impairment in another. For example, a man with normal concentration may still have erectile dysfunction, sperm DNA fragmentation, endocrine disease, or obstruction; conversely, low sperm count does not establish the cause without clinical context (2, 4). A broad framework therefore prevents reduction of male reproductive assessment to a single laboratory number (1, 9).

Infertility affects individuals, couples, and communities. WHO estimates that about 17.5% of adults experience infertility during their lifetime, equivalent to roughly one in six people (1, 12). Male factors may occur alone, alongside female factors, or in combined and unexplained infertility; the estimate should never be used to assign blame (1, 13).

The public-health dimension reaches beyond conception. Infertility can be associated with stigma, relationship strain, economic hardship, social exclusion, and unregulated use of medicines, supplements, or traditional preparations (14, 15). Men may delay assessment because they expect judgement or assume infertility is exclusively a female concern, allowing treatable conditions and fertility-preservation opportunities to be missed (1, 2).

Population-level concern has also been shaped by evidence of temporal changes in sperm count. Levine et al. (16) reported a 51.6% decline in mean sperm concentration across samples collected between 1973 and 2018, with a steeper estimated decline in analyses restricted to samples collected after 2000. This estimate should be interpreted as a population-level temporal signal rather than a direct measure of individual infertility. Its interpretation is constrained by changes in recruitment frames, laboratory counting methods and quality control, abstinence reporting, geographic coverage, and other sources of between-study heterogeneity across five decades; therefore, temporal association alone cannot identify a specific causal exposure (3, 16, 17).

Environmental, pharmacological, and disease-related influences are frequently evaluated in separate research and clinical domains, although affected individuals may experience several simultaneously. For example, obesity and type 2 diabetes may coexist with antihypertensive therapy, occupational heat, traffic-related air pollution, and sleep disruption. These factors can converge on endocrine, inflammatory, vascular, and spermatogenic pathways, making cumulative exposure and comorbidity clinically relevant while complicating causal attribution (1, 2). An integrated evidence map is therefore appropriate for identifying overlapping pathways, areas of stronger evidence, and unresolved research gaps.

The evidence base requires careful interpretation. Animal and mechanistic studies establish biological plausibility, whereas observational human studies can identify associations but remain vulnerable to exposure misclassification, confounding, and laboratory variation (10, 18). Modifiable risks should therefore be reduced where feasible, but no patient should be told that a single exposure certainly caused infertility without a complete clinical assessment (1, 2).

Causal inference is particularly difficult for environmental exposures because randomised trials that deliberately expose people to toxicants are unethical. Repeated biomarker measurement, prospective preconception designs, standardised semen laboratories, and outcomes that include pregnancy and live birth can strengthen future evidence (16, 18).

Accordingly, this scoping review maps how environmental exposures, pharmacological agents and substance use, and disease conditions are associated with male reproductive health; identifies convergent mechanisms; describes current approaches to assessment and prevention; and highlights evidence gaps relevant to research, clinical practice, and public health.

## 2 Methods

### 2.1 Review design and reporting framework

A scoping-review design was selected because the review question is broad, spans heterogeneous evidence types, and aims to map concepts, evidence characteristics, mechanisms, clinical implications, and knowledge gaps rather than estimate a single pooled intervention effect. The review was structured using JBI methodological guidance for scoping reviews and reported with reference to PRISMA-ScR; reporting of electronic searches was informed by PRISMA-S (19–22). These four methodological references informed review conduct and reporting but were not counted among the 105 evidence sources mapped in **Figure 1**.

**Figure 1:**
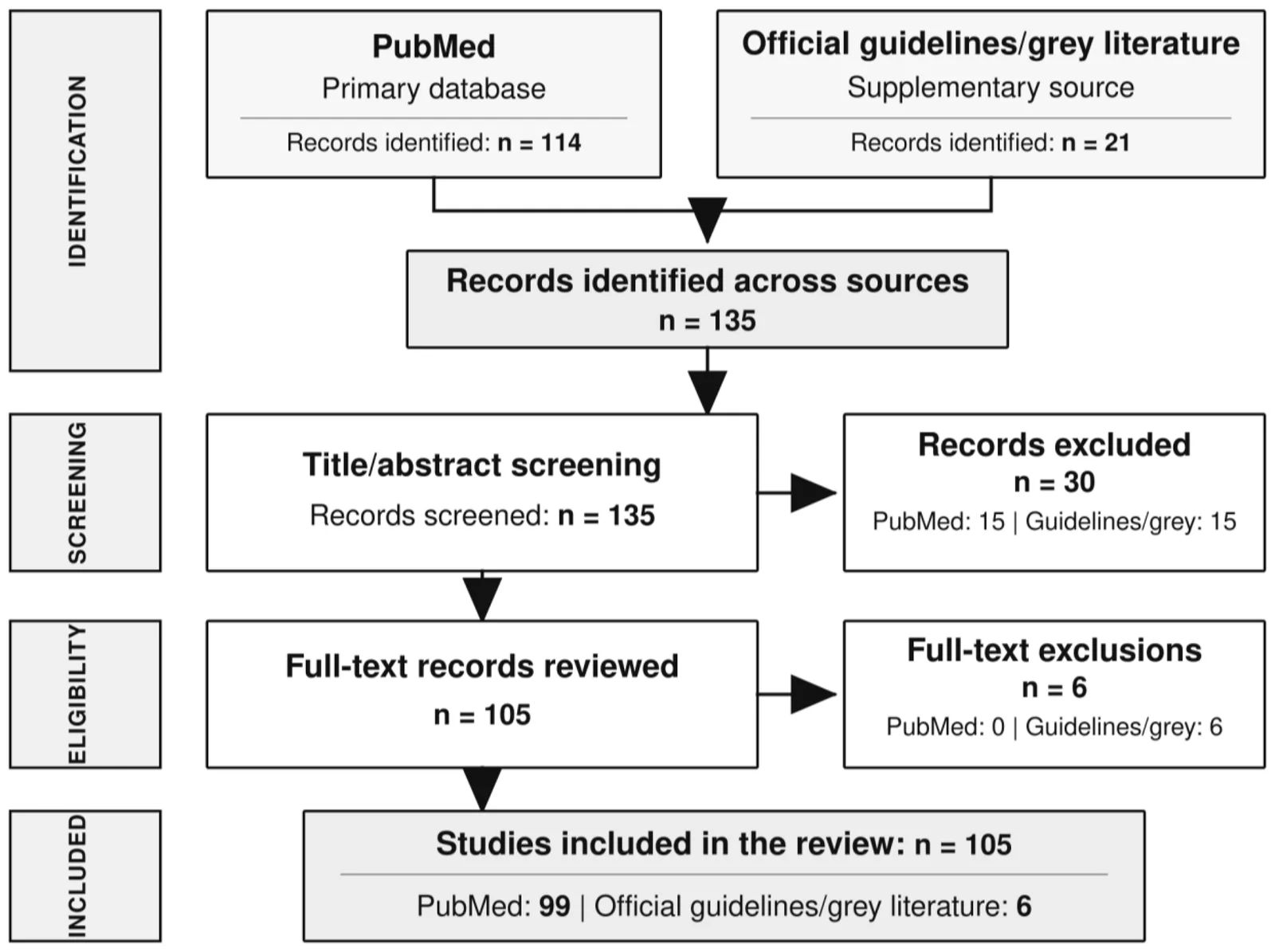
PRISMA-ScR flow of source identification and evidence mapping. The evidence map contains 105 sources; four methodological references are reported separately and are not included in the mapped evidence count.

### 2.2 Review question and eligibility framework

Eligibility was organised using the Population-Concept-Context framework. The population comprised males across reproductive and adult age groups where a male reproductive outcome was reported. The concept comprised environmental exposures, medicines and other pharmacological agents, substance use, acute and chronic diseases, and their biological or clinical relationships with spermatogenesis, semen quality, reproductive hormones, sexual or ejaculatory function, sperm DNA integrity, fertility, or fertility-preservation decisions. The context was unrestricted by country, health-care setting, or occupational setting because the purpose was to map the global evidence base and identify context-specific limitations. The principal review question was: What evidence describes how environmental, pharmacological, and disease-related influences affect male reproductive health, through which mechanisms, and with what implications for assessment, prevention, and management?

### 2.3 Information sources and search date

PubMed was the primary bibliographic database. Google Scholar and official grey literature/guidelines were supplementary sources. Searches covered database inception to August 2026. Google Scholar supported multidisciplinary discovery and forward/backward citation chaining, while official guidance was sought from WHO, the European Association of Urology (EAU), AUA/ASRM, ASCO, and the National Institute for Health and Care Excellence (NICE). The mapped evidence set comprised 99 PubMed-indexed peer-reviewed sources and six official grey-literature/guideline sources, for 105 unique evidence sources. Four additional methodological references were used to guide scoping-review conduct and reporting and were not included in the evidence-map count.

### 2.4 Inclusion and exclusion criteria

Included sources were: (1) human observational or interventional studies reporting male reproductive outcomes; (2) systematic reviews, meta-analyses, and scoping reviews relevant to the mapped domains; (3) clinical practice guidelines or consensus statements from recognised professional or public-health organisations; and (4) selected animal or in-vitro mechanistic studies only where they clarified pathways not directly measurable in humans, particularly epigenetic or toxicological mechanisms. Excluded sources were studies without a male reproductive outcome, non-reproductive ecotoxicology studies, duplicate reports of the same evidence without additional information, conference abstracts or opinion pieces lacking sufficient methodological detail, and sources whose claims could not be linked to the review question. No geographic restriction was applied. English-language sources were used for the present synthesis, and recent higher-level evidence was prioritised while older seminal evidence was retained where still relevant to current guidance.

### 2.5 Evidence selection and bibliographic verification

Records were assessed against the Population-Concept-Context eligibility framework and verified at full-record or full-text level before evidence charting. PubMed records served as the primary bibliographic anchors; Google Scholar, publisher pages, and official organizational websites were supplementary. The evidence map contains 105 unique sources: 99 peer-reviewed journal records indexed in PubMed and six official grey-literature/guideline sources. No journal record was counted twice merely because it was discoverable through both PubMed and Google Scholar. The complete search strategy and source-level evidence map are provided in **Supplementary Tables S1** and **S2**, respectively.

### 2.6 PRISMA-ScR flow and selection transparency

**Figure 1** presents the PRISMA-ScR source-identification and evidence-mapping flow. The mapped synthesis contains 105 unique evidence sources: 99 PubMed-indexed peer-reviewed records and six official grey-literature/guideline sources. Google Scholar was used as a supplementary discovery and citation-chaining route; overlapping PubMed records were counted once after de-duplication. Four methodological references used to guide scoping-review conduct and reporting were not included in the 105-source evidence map and bring the complete bibliography to 109 references.

### 2.7 Data charting and synthesis

For each retained evidence source, the synthesis charted the citation, publication year, study or evidence type, population or exposure context, reproductive endpoint, principal quantitative result where reported, proposed mechanism, major limitation, and clinical or public-health implication. Evidence was then organised into environmental, pharmacological, disease-related, mechanistic, diagnostic, preventive, and policy domains. No de novo meta-analysis was undertaken; quantitative estimates are reported only when they originate from cited primary studies or published meta-analyses. Given the mapping objective, no single cross-design quality score was imposed. Instead, the interpretation distinguishes guideline evidence, meta-analytic evidence, observational associations, and mechanistic evidence and explicitly identifies uncertainty, confounding, and exposure-measurement limitations.

### 2.8 Reproducible search strategy

The exact PubMed Boolean strategies and supplementary Google Scholar search logic are provided in **Supplementary Table S1**. The complete source-level evidence map, including APA author–date citations, study design or sample information, and quantitative or evidential contributions, is provided in **Supplementary Table S2**.

## 3 Overview of normal male reproductive function

**Table 1** provides a quantitative overview of the principal evidence anchors identified across the scoping review, allowing the magnitude, direction, and certainty of findings from different domains to be considered together. It shows relevant evidence syntheses covering environmental exposures, including pesticides, potentially toxic elements, air pollution, heat, endocrine-disrupting chemicals, and tobacco smoke; metabolic and disease-related factors, including obesity, diabetes, varicocele, and genital-tract infections; pharmacological influences, including clomiphene, valproate, antidepressants, and treatments for idiopathic infertility; and diagnostic or population-level outcomes, including sperm DNA fragmentation and temporal trends in sperm concentration. Where available, effect estimates such as standardised mean differences, mean differences, odds ratios, regression coefficients, confidence intervals, and study or participant numbers are presented alongside interpretive cautions concerning heterogeneity, observational designs, exposure measurement, and certainty of evidence. This quantitative evidence map therefore provides an empirical foundation for the subsequent sections, while Sections 3.1–3.4 focus specifically on the normal anatomical, endocrine, spermatogenic, and sexual-function processes

**Table 1:** Quantitative evidence anchors across environmental, pharmacological, disease, biomarker, and population domains.

Domain	References	Design/sample	Quantitative finding	Possible interpretation
Temporal trend	Levine et al. (16)	Systematic review/meta-regression	51.6% decline in mean sperm concentration across samples collected 1973-2018.	Population-level temporal signal; not proof of a specific causal exposure.
Pesticides	Giulioni et al. (23)	Systematic review; 64 human studies	Recurring adverse findings for semen parameters and sperm DNA/chromatin integrity.	Heterogeneity by pesticide class and study design.
Pyrethroids	Akhigbe et al. (24)	Meta-analysis; 12 studies, 2,050 men	Lower sperm motility overall; regional variation for count/concentration.	Association with substantial heterogeneity.
Potentially toxic elements	Kaur et al. (25)	Systematic review/meta-analysis	Blood lead SMD 0.67; seminal lead 1.19; seminal cadmium 0.92.	Supports metal-specific rather than blanket inference.
Smoking	Sharma et al. (26)	Meta-analysis; 20 studies, 5,865 men	Count MD -9.72×10^6/mL; motility -3.48%; morphology -1.37%.	Greater effects in infertile and moderate/heavy smokers.
Air pollution	Zhao et al. (27)	Cohort; 33,876 men, 340 Chinese cities	PM2.5/PM10 exposure associated with lower total/progressive motility and more asthenozoospermia.	Observational; exposure-model limitations remain.
Air pollution	Margiana et al. (28)	Meta-umbrella of 4 meta-analyses	Total motility SMD -0.24; progressive motility -0.06; morphology -0.09.	Modest pooled effects; exposure thresholds uncertain.
Heat	Hoang-Thi et al. (29)	Meta-analysis; 9 studies, 356 men	Concentration SMD -1.07; motility SMD -1.93; several other semen parameters also lower.	Supports occupational heat prevention; causality for climate trends not established.
Obesity	Li et al. (30)	Meta-analysis; 50 studies, 71,337 subjects	Obesity associated with -0.24 mL semen volume, -19.56×10^6 total sperm, -2.21% total motility, -5.95% progressive motility, -1.08% normal forms.	Associations are pooled and heterogeneous.
Weight loss	Santi et al. (31)	Meta-analysis; 12 studies, 345 men	Improvement in sperm concentration, progressive motility and DNA fragmentation.	Supports sustainable metabolic management.
Varicocele repair	Cannarella et al. (32)	Meta-analysis; 351 studies (23 RCTs, 292 observational, 36 case-control)	After repair: sperm concentration SMD 1.590 (95% CI 1.474–1.706); total count 1.824 (1.526–2.121); progressive motility 1.845 (1.537–2.153); morphology 1.066 (0.992–1.211).	Large evidence base, but I² was approximately 98% for several endpoints and publication bias was detected for some outcomes.
Varicocele pregnancy	Wang et al. (33)	Meta-analysis; 4 RCTs, 349 participants	No statistically clear improvement in spontaneous pregnancy after primary surgery.	Supports selective repair and shared decision-making.
Idiopathic infertility treatment	Melhem et al. (34)	Meta-analysis; 41 RCTs, 3,615 men	Antioxidant concentration SMD 1.09; progressive motility 1.11; total motility 0.69; morphology 0.43; prediction intervals crossed null.	Very-low-certainty evidence; avoid indiscriminate empirical therapy.
Antidepressants	Wang et al. (35)	Network meta-analysis; 30 RCTs, 18,157 participants	EjD risk versus placebo: clomipramine OR 42.11 (95% CI 9.90–179.08), WS5570 OR 28.99 (1.48–568.97), paroxetine OR 18.63 (9.33–37.23).	Sexual adverse effects vary substantially by antidepressant; psychiatric efficacy and treatment continuity must also be considered.
African sperm-count trend	Sengupta et al. (36)	Meta-analysis; 14 studies	Reported 72.6% decrease in mean sperm concentration from 1965-2015.	Historical heterogeneity and sparse regional evidence require cautious interpretation.
Endocrine disruptors/BPA	Lü et al. (37)	Systematic review/meta-analysis; 18 studies	Urinary BPA associated with sperm concentration β=-0.03 (95% CI -0.06 to -0.01) and total sperm count β=-0.05 (95% CI -0.08 to -0.02).	Statistically significant pooled associations; below-reference semen-quality associations were not consistently robust.
Varicocele/endocrine	Ziouziou et al. (38)	Systematic review/meta-analysis; 32 studies	FSH MD 2.18 IU/L; LH MD 0.63 IU/L; total testosterone MD -0.71 ng/mL in men with varicocele.	Supports endocrine as well as semen effects; does not alone determine surgical candidacy.
Genital-tract infection	Campbell et al. (39)	Systematic review/meta-analysis	HPV-antisperm antibody OR 10.63 (95% CI 1.49-75.93); HBV-miscarriage OR 1.43 (95% CI 1.05-1.93).	Most pregnancy/live-birth outcomes were nonsignificant; substantial heterogeneity.
Sperm DNA fragmentation	Vitthala et al. (40)	Systematic review/meta-analysis; 52 studies, 19,930 subjects	High SDF showed low sensitivity and higher specificity with poor-to-moderate overall discrimination.	Assay-dependent thresholds and heterogeneity limit universal screening.
Type 1 diabetes	Facondo et al. (41)	Systematic review/meta-analysis; 14 studies; 8 semen studies meta-analysed	DM1 (n=380) vs controls (n=434): normal morphology MD −0.36 percentage points (95% CI −0.66 to −0.06); progressive motility MD −33.62 percentage points (95% CI −39.13 to −28.11).	Selected semen endpoints were impaired; hormonal and broader fertility outcomes remain heterogeneous.
Clomiphene	Huijben et al. (42)	Systematic review/meta-analysis; 18 studies, 731 men; 15 studies/566 in meta-analysis	Sperm concentration MD + 8.38×10^6/mL (95% CI 5.17–11.59); total motility MD + 8.14 percentage points (95% CI 3.83–12.45); mean pregnancy rate 17% across 10 studies.	Potential benefit in selected men; high heterogeneity and off-label use require specialist assessment.
Male adiposity	Salas-Huetos et al. (43)	Systematic review; 60 qualitative and 28 quantitative studies	Overweight/obesity associated with lower semen volume, sperm count/concentration, vitality, total motility and normal morphology and with adverse reproductive-hormone profiles.	Supports adiposity as a modifiable risk context; pooled outcomes are heterogeneous.
Industrial air pollution	Ramsay et al. (44)	Retrospective cohort; 21,563 men	Residential exposure to industrial air emissions was associated with poorer semen/fertility outcomes.	Large cohort, but observational exposure modelling limits causal attribution.
Phthalate mixtures	Mizuno et al. (45)	Cross-sectional study; 197 men	WQS, q-g-computation and BKMR identified mixture associations with motility and reproductive hormones; no significant association with volume, count or concentration.	Shows why mixture analysis can yield multidirectional associations and requires careful causal interpretation.
Sperm DNA fragmentation	Esteves et al. (46)	Evidence synthesis/practice recommendations	41 recommendations: 13 technical/testing and 28 indication/management recommendations.	Supports selective SDF testing; evidence quality was largely moderate to low.
Global infertility prevalence	World Health Organization (12)	Global pooled prevalence estimates	Lifetime infertility prevalence estimated at approximately 17.5% of adults, about 1 in 6.	Population prevalence; does not quantify male-factor contribution in an individual couple.
Organophosphate pesticides	Hamed et al. (47)	Systematic review/meta-analysis; 766 men (349 exposed, 417 controls)	Exposure was associated with lower sperm concentration, total motility, and normal morphology; sperm count and progressive motility were not robust after all sensitivity analyses; FSH, LH, and testosterone were not significantly different.	Supports a semen-quality association while showing endpoint sensitivity and no clear testosterone-mediated effect.
Ambient air pollution	Xu et al. (48)	Systematic review/meta-analysis; 11 studies	Each 10 μg/m³ increase in PM10 was associated with 2.18% lower sperm concentration and 2.76% lower total sperm number; SO2 with 8.61% lower concentration and 9.52% lower total number; PM2.5 and PM10 were associated with lower total/progressive motility.	Provides exposure-response estimates across several pollutants; gaseous-pollutant and morphology evidence was less consistent.
Valproate	Asghar et al. (49)	Meta-analysis; 5 clinical studies, 274 men (plus 7 preclinical studies)	Clinical sperm motility SMD −1.62 (95% CI −2.81 to −0.43); sperm count SMD −0.78 (−1.58 to 0.03) was not statistically significant; FSH and LH differences were also nonsignificant.	Human evidence suggests a motility effect but remains based on a small clinical evidence base.

### 3.1 Anatomy of the male reproductive system

The testes have two core functions: sperm production within the seminiferous tubules and testosterone synthesis by interstitial Leydig cells (6, 7). Sertoli cells support germ-cell development, form the blood-testis barrier, regulate the tubular environment, and release inhibin B (7, 50). Sperm then pass through the epididymis for maturation, the vas deferens for transport, and accessory-gland secretions that contribute to semen volume, pH, liquefaction, and motility support (6, 9).

The anatomical route also has direct diagnostic value. Low-volume acidic semen, absent vasa deferentia, epididymal fullness, small testes, or a palpable varicocele may each point to different mechanisms and different next investigations (2, 9). This is why semen analysis, examination, and endocrine assessment are complementary rather than interchangeable (6, 8).

### 3.2 Hormonal regulation of male reproduction

Male reproductive endocrinology is governed by the hypothalamic-pituitary-gonadal axis. Pulsatile gonadotropin-releasing hormone stimulates luteinising hormone and follicle-stimulating hormone, which respectively support Leydig-cell testosterone production and Sertoli-cell-mediated spermatogenesis (2, 6). Systemic illness, obesity, sleep disruption, opioid exposure, hyperprolactinaemia, exogenous androgens, pituitary disease, and caloric extremes can perturb this axis (51, 52).

A single low testosterone result does not establish hypogonadism. Testosterone exhibits diurnal variation, can fall transiently during acute illness, and should be interpreted with symptoms, repeat morning testing, sex hormone-binding globulin where relevant, luteinising hormone, follicle-stimulating hormone, prolactin, and clinical context (1, 2). Equally, a serum testosterone level within a laboratory reference interval does not guarantee normal spermatogenesis. Intratesticular testosterone concentrations, Sertoli-cell function, genetic integrity, temperature, and epididymal maturation all matter (1, 2). This distinction is vital when counselling men who seek testosterone therapy while hoping to conceive, because exogenous testosterone can suppress rather than enhance sperm production (2, 51).

For men with confirmed hypogonadal symptoms who wish to preserve fertility, exogenous testosterone should not be used as an empirical fertility treatment. Specialist evaluation should first establish whether the condition is primary or secondary and identify reversible causes. In selected men, fertility-preserving approaches may include gonadotropin therapy for hypogonadotropic hypogonadism or off-label agents that stimulate endogenous hypothalamic-pituitary-gonadal signalling, such as selective oestrogen receptor modulators. Evidence for clomiphene shows improvement in sperm concentration and motility across heterogeneous studies, but treatment selection, dosing, monitoring, and expectations should remain specialist-led rather than presented as universally effective (9, 42, 53).

### 3.3 Spermatogenesis and semen quality

Spermatogenesis is a continuous developmental process involving spermatogonial proliferation, meiosis, spermiogenesis, epididymal maturation, and transport. Its duration means that fever, toxic exposure, medication change, or metabolic deterioration may not appear immediately in an ejaculate (6, 9). Repeated semen analysis after a modifiable insult is usually more informative than treating one sample as a permanent verdict (4, 50).

Semen analysis remains the central laboratory assessment. The WHO sixth edition lists lower fifth-centile reference values from fertile men of 1.4 mL for volume, 16 million/mL for concentration, 39 million for total sperm number, 42% for total motility, 30% for progressive motility, and 4% for normal morphology (6, 50). These are reference limits rather than sharp boundaries between fertility and infertility, because conception also depends on sperm DNA integrity, sexual function, timing, and partner factors (8; 4).

Laboratory quality assurance is essential because collection conditions, reading methods, staff training, and reporting conventions influence apparent differences between samples and centres. Repeat testing in a validated laboratory is often more clinically useful than extensive interpretation of one borderline result (6, 50).

Sperm DNA fragmentation and oxidative stress are clinically relevant but require selective use. Elevated fragmentation has been associated with poorer pregnancy outcomes, recurrent pregnancy loss, unexplained infertility, and some adverse assisted-reproduction outcomes (46). Methods and thresholds differ, so testing is best considered in selected settings rather than applied universally (2, 9).

A 2026 systematic review and meta-analysis included 52 studies and 19,930 subjects. High sperm DNA fragmentation showed low sensitivity and higher specificity across natural and assisted conception, with poor-to-moderate overall diagnostic discrimination, assay-specific cut-offs, and substantial heterogeneity (40). These findings support selective rather than universal use and interpretation alongside clinical context and assay methodology.

When fragmentation is elevated, the clinical priority is to search for potentially modifiable contributors such as varicocele, tobacco use, febrile illness, genital inflammation, obesity, or occupational toxicants. Empirical antioxidant use alone should not replace diagnosis, exposure control, or treatment of a defined condition (46).

### 3.4 Sexual function and male reproductive health

Sexual function is integral to male reproductive health. Erection requires adequate arterial inflow, endothelial nitric-oxide signalling, relaxation of cavernosal smooth muscle, venous occlusion, autonomic and sensory neural pathways, psychological security, and an appropriate hormonal milieu (1, 2). Ejaculation requires coordinated sympathetic, somatic, and pelvic-floor activity. Disorders of desire, erection, ejaculation, orgasm, or pain can prevent conception even in the presence of satisfactory semen parameters (1, 2). They may be caused by diabetes, cardiovascular disease, neurological injury, pelvic surgery, medications, depression, relationship stress, substance use, or fear of infertility itself.

Sexual dysfunction can prevent conception even when conventional semen parameters are satisfactory, and fertility difficulties can in turn reduce sexual confidence and intercourse frequency. Assessment should therefore include desire, erections, ejaculation, pain, relationship factors, and mental health rather than assuming that semen quality alone determines the pathway to pregnancy (1, 54).

Erectile dysfunction is also a potential marker of wider cardiometabolic risk. The Princeton IV consensus recommends that men presenting with erectile dysfunction be evaluated for cardiovascular risk because vascular and endothelial dysfunction may precede overt cardiovascular events (54). This does not imply that every episode of erectile difficulty indicates heart disease. It means that sexual symptoms offer a clinically valuable opening to assess blood pressure, glycaemic status, lipids, smoking, obesity, sleep, medication effects, and emotional wellbeing (1, 2). Reproductive care gains quality when sexual health is assessed without embarrassment or moral judgement.

## 4 Environmental influences on male reproductive health

Environmental exposures can affect male reproductive health through oxidative stress, endocrine disruption, inflammation, mitochondrial injury, and altered testicular or sperm function, although individual causal attribution remains difficult in most human studies (10, 18). The most important exposure categories include endocrine-disrupting chemicals, pesticides, heavy metals, air pollutants, occupational heat, ionising radiation, tobacco smoke, and mixed occupational or household exposures (47, 48, 55). **Table 2** summarises the major environmental exposure groups, the available evidence, plausible reproductive mechanisms, and the corresponding clinical and public-health implications.

**Table 2:** Environmental exposures, evidence interpretation, mechanisms, and implications for male reproductive health.

Exposure group	Human evidence	Plausible reproductive pathway	Clinical and public-health implication
Endocrine-disrupting chemicals (bisphenols, phthalates, PFAS, persistent organics)	Human biomarker studies report heterogeneous associations of bisphenols and phthalates with semen concentration, motility, morphology, or reproductive hormones; short-lived urinary biomarkers and co-exposure confounding limit individual causal attribution (55–58).	Interference with steroidogenesis, receptor signalling, Sertoli/Leydig-cell function, oxidative stress, and developmental programming.	Reduce avoidable exposure, especially occupational or food-contact heating exposures. Do not attribute infertility to one biomarker alone; evaluate co-exposures and clinical causes.
Pesticides and herbicides	Systematic reviews consistently identify pesticide exposure as a reproductive-health concern, although effect sizes vary by compound and study design. A review of 64 human studies reported recurring adverse findings for semen parameters and sperm DNA/chromatin integrity (23). A 2024 meta-analysis of 12 cross-sectional studies involving 2,050 men found significantly lower sperm motility overall, with region-specific changes in count and concentration and substantial heterogeneity (24). Organophosphate-focused evidence likewise reports lower count, concentration, motility, and morphology in pooled analyses, with sensitivity-related variation across endpoints (47).	Oxidative injury, mitochondrial dysfunction, germ-cell apoptosis, altered steroidogenic signalling.	Prioritise workplace controls: substitution, training, PPE, hygiene, re-entry intervals, and occupational-health review during conception planning.
Heavy metals: lead, cadmium, mercury, arsenic	Human evidence is mainly observational and exposure-specific. Reproductive toxicology literature repeatedly identifies metals as plausible risks in mining, e-waste, welding, tobacco, battery, and contaminated-water settings (10).	Blood-testis barrier disruption, reactive oxygen species, mitochondrial injury, impaired testosterone synthesis, DNA damage.	Identify source and involve occupational/environmental health services; generic supplements are not a substitute for source control.
Ambient particulate air pollution	Meta-analysis found adverse associations with semen outcomes (48). A cohort of 33,876 men linked PM2.5/PM10 exposure with lower motility and more asthenozoospermia (27).	Systemic inflammation, oxidative stress, endocrine perturbation, possible direct testicular effects.	Counsel on avoiding added smoke and uncontrolled workplace exposure; advocate emissions control and clean indoor energy.
Heat and high-temperature work	A meta-analysis of nine studies involving 356 men found significantly lower semen volume, concentration, total count, motility, progressive motility, and morphology under high-temperature exposure; pooled SMDs included -1.07 for concentration and -1.93 for motility (29).	Elevated scrotal temperature disrupts spermatogenesis and increases oxidative stress.	Improve ventilation, work-rest cycles, hydration, job rotation, and management of varicocele or recurrent fever; reassess after a spermatogenic interval.
Ionising radiation	Established gonadotoxic potential when testes/pituitary are within the treatment field or cumulative exposure is significant.	Direct DNA injury and depletion of dividing germ cells; possible endocrine damage.	Provide pre-treatment fertility counselling and sperm cryopreservation before relevant cancer therapy (59).
Tobacco smoke, heavy alcohol, and mixed social exposures	Consistent public-health concern; exposure often coexists with poor sleep, metabolic risk, and occupational hazards.	Oxidative stress, vascular dysfunction, endocrine effects, liver injury, impaired sexual function.	Offer cessation and dependence support without stigma; assess social conditions that constrain behaviour change.
Traffic-related hydrocarbons and industrial air mixtures	Systematic reviews and cohorts support possible adverse associations between mixed air pollutants and semen quality, but exposure models differ across settings (44, 60–62).	Oxidative stress, systemic inflammation, endocrine disturbance, and possible testicular toxicant transport.	Control workplace emissions, reduce unnecessary combustion exposure, and treat population-level emission control as reproductive-health policy.
E-cigarette aerosol and novel nicotine exposure	Direct human fertility evidence remains limited. Toxicology and systematic-review evidence supports precaution because aerosol constituents may not be biologically inert (63).	Oxidative stress, metal/carbonyl exposure, endothelial and endocrine effects.	Do not present e-cigarettes as a proven fertility-safe alternative; support nicotine-dependence treatment.

Semen parameters are intermediate markers and cannot, by themselves, determine a man’s ability to achieve a pregnancy or establish an individual causal attribution. Evidence categories in this table distinguish biological plausibility from proven individual causation.

### 4.1 Environmental pollution and reproductive toxicity

Recent high-level evidence strengthens several parts of this map. A systematic review of environmental and occupational exposures identified smoking, obesity, diabetes and exposure to endocrine-disrupting chemicals such as phthalates, bisphenols and pesticides as recurring male-fertility concerns across studies published from 2000 to 2020 (64). A separate systematic review of polycyclic aromatic hydrocarbons reported consistent adverse associations with semen volume, concentration, motility, morphology and sperm DNA integrity, while emphasizing heterogeneity across exposure metrics and study designs (65).

Environmental reproductive toxicology considers exposures encountered through air, water, food, soil, consumer products, housing, transport, and employment that may impair reproductive development or function. Exposure may occur by inhalation, ingestion, dermal absorption, or transplacental transfer (1, 2). In adult men, studies often examine urinary or blood biomarkers alongside semen parameters or hormones. Developmental exposure is also relevant because fetal and pubertal windows may alter testicular differentiation, Sertoli-cell number, endocrine signalling, and later reproductive potential (1, 2). A lifetime perspective is more realistic than an assumption that fertility is affected only by what is encountered during the month of semen testing (10, 66).

Exposure assessment should distinguish source, route, duration, intensity, timing, and co-exposures. A urinary biomarker may reflect recent contact, whereas testicular development and spermatogenesis can be influenced by earlier life stages or by repeated occupational exposure that is not captured by one measurement (10, 66).

Mixed-exposure epidemiology is particularly challenging because environmental chemicals and social or metabolic exposures are correlated, may share sources, can act non-linearly, and are often measured with different biological half-lives. Single-pollutant regression can therefore obscure joint effects or produce unstable estimates when exposures are highly correlated. Mixture-oriented approaches such as weighted quantile sum regression, quantile g-computation, and Bayesian kernel machine regression can estimate overall mixture effects and identify influential components while accommodating correlation or non-linearity under different assumptions. Their results remain model-dependent and require careful interpretation; they strengthen mixture assessment but do not remove exposure measurement error, residual confounding, or the need for replication (18, 45).

### 4.2 Endocrine-disrupting chemicals

Endocrine-disrupting chemicals are exogenous agents that can interfere with hormone synthesis, metabolism, transport, receptor binding, or feedback regulation (55, 66). Bisphenols, phthalates, parabens, alkylphenols, persistent organics, flame retardants, and PFAS can occur in plastics, food-contact materials, personal-care products, dust, textiles, coatings, and industrial processes (67, 68). Experimental evidence for testicular and steroidogenic effects is often stronger than causal human evidence, which varies by exposure timing and measurement (11, 69).

The developmental window matters because fetal and pubertal endocrine disruption may alter testicular differentiation, Sertoli-cell number, and later reproductive potential. Adult cross-sectional studies remain useful, but they cannot fully reconstruct those earlier exposures or explain all interindividual susceptibility (66, 69).

Bisphenol A illustrates the evidence challenge. Experimental models support estrogenic and anti-androgenic activity, while epidemiological studies have reported associations with semen concentration, motility, morphology, and reproductive hormones (56, 57). The human literature remains heterogeneous and does not establish causal risk for every individual, so counselling should prioritise practical exposure reduction rather than single-product promises (55, 58).

A 2024 systematic review and meta-analysis of 18 epidemiological studies found inverse associations between urinary BPA and sperm concentration (β=-0.03, 95% CI -0.06 to -0.01) and total sperm count (β=-0.05, 95% CI -0.08 to -0.02), together with associations involving SHBG, estradiol and biologically active androgen measures. However, associations with below-reference semen quality were not consistently robust, reinforcing the need for cautious causal interpretation (37).

### 4.3 Pesticides and herbicides

Agricultural workers, pesticide applicators, farmers, greenhouse workers, and communities near spraying areas may experience repeated contact with organophosphates, carbamates, pyrethroids, herbicides, fungicides, and mixed formulations. Exposure may occur during mixing, spraying, equipment cleaning, field re-entry, or handling contaminated crops and clothing (23, 70–72).

The human evidence is strongest for an association rather than individual causal attribution. Giulioni et al. (23) synthesised 64 human studies and found recurring adverse findings for semen parameters and sperm DNA/chromatin integrity, particularly in relation to organophosphate, organochlorine, and pyrethroid exposure. In a 2024 systematic review and meta-analysis of 12 cross-sectional studies involving 2,050 men, Akhigbe et al. (24) reported significantly lower sperm motility overall, while count and concentration findings varied by region and the authors emphasised inconsistency and heterogeneity. Organophosphate-focused meta-analysis also reported lower sperm count, concentration, motility, and normal morphology across included studies, although not every endpoint remained robust in sensitivity analyses (47). Proposed pathways include oxidative stress, mitochondrial dysfunction, germ-cell apoptosis, altered steroidogenesis, and DNA damage (10, 73).

Prevention should therefore extend beyond individual counselling. Substitution of hazardous products, closed mixing systems, engineering controls, correct dilution, training, suitable protective equipment, hygiene facilities, and enforcement of re-entry intervals can reduce exposure more reliably than relying on worker behaviour alone (1, 72).

### 4.4 Heavy metals

A 2024 systematic review and meta-analysis provides quantitative support for selected metals. Compared with fertile controls, infertile men had higher blood lead (SMD 0.67, 95% CI 0.07-1.28), higher seminal lead (SMD 1.19, 95% CI 0.42-1.96), and higher seminal cadmium (SMD 0.92, 95% CI 0.54-1.29); pooled associations for arsenic, barium and mercury in blood were not statistically significant (25). These results support source-specific exposure assessment rather than treating all metals as having equivalent evidence.

Lead, cadmium, mercury, arsenic, chromium, and other metals can reach workers and communities through mining, battery recycling, e-waste, welding, contaminated water, tobacco smoke, traffic emissions, and industrial dust. Human reproductive evidence remains largely observational and exposure-specific, but toxicological and clinical reviews support plausible effects on the blood-testis barrier, mitochondrial function, Leydig-cell steroidogenesis, oxidative stress, and germ-cell survival (10, 74).

Because metal toxicokinetics differ, the exposure source should guide investigation. A man who repairs batteries, recycles electronics, welds in poorly ventilated settings, or handles contaminated soil may need occupational-health review and local biomonitoring protocols rather than generic supplement advice (10, 74).

### 4.5 Air pollution

Ambient air pollution is increasingly relevant because exposure is widespread and often involuntary. Particulate matter, nitrogen oxides, ozone, sulphur dioxide, traffic-related hydrocarbons, and industrial emissions may promote systemic inflammation and oxidative stress that affect endocrine and testicular function. Systematic reviews and cohort studies report adverse associations with semen outcomes, although pollutant metrics, spatial models, and exposure windows differ across studies (44, 48, 61). A 2026 meta-umbrella analysis of meta-analytic evidence likewise reported modest adverse associations between higher air-pollution exposure and several semen-quality outcomes, while emphasizing remaining uncertainty in magnitude and exposure thresholds (28).

A cohort study of 33,876 men from 340 Chinese cities associated PM2.5 and PM10 exposure with lower total and progressive motility and a higher risk of asthenozoospermia during relevant spermatogenic windows (27). The result strengthens concern but remains observational, as activity patterns, indoor exposures, smoking, occupational conditions, and spatial modelling can influence estimates (10, 48).

At individual level, reducing added smoke, improving ventilation where indoor combustion occurs, and using appropriate workplace protection may be sensible. However, such measures cannot replace population-level controls on emissions, transport, energy sources, and industrial pollution, which are the more equitable route to risk reduction (1, 68).

### 4.6 Heat exposure and thermal stress

The location of the testes outside the abdominal cavity supports a temperature slightly below core body temperature that is important for spermatogenesis. Sustained scrotal heat can increase oxidative stress and disturb germ-cell maturation, with relevance to foundries, kitchens, boiler rooms, long-distance driving, mining, and heat-retaining protective clothing (9, 10). Clinical advice should focus on repeated high-heat exposure, fever, and varicocele rather than attributing severe infertility to occasional warm bathing (2, 11).

Climate-related heat exposure is an emerging reproductive-health concern because background ambient temperature and occupational heat can overlap. A 2022 meta-analysis of nine studies involving 356 men reported significantly lower semen volume, sperm concentration, total sperm count, motility, progressive motility, and normal morphology in high-temperature exposure groups; for example, the pooled standardised mean differences were -1.07 for sperm concentration and -1.93 for motility (29). These findings support heat-risk prevention and further longitudinal research, but they should not be interpreted as proof that climate change alone explains population fertility trends because occupation, acclimatisation, seasonality, health status, and exposure measurement vary substantially.

### 4.7 Radiation and electromagnetic exposure

Ionising radiation can damage DNA and dividing germ cells. Risk depends on dose, fractionation, radiation field, shielding, age, baseline testicular reserve, and concurrent chemotherapy (59, 75). A fertility-preservation discussion should occur before relevant oncological radiation, particularly where the testes, pelvis, pituitary, or whole body may be exposed (2, 59).

In oncology settings, testicular shielding, treatment-field planning, and coordination between oncology and fertility teams can reduce avoidable harm while preserving treatment effectiveness. The urgency and prognosis of cancer treatment remain central, so reproductive decisions must be made promptly and without pressure to delay essential care (1, 59).

Evidence about non-ionising electromagnetic exposure from consumer devices is less conclusive because actual personal exposure is difficult to measure and human studies are heterogeneous. It is reasonable to avoid prolonged local heating of the scrotum, but claims that routine wireless-device use is a proven major cause of infertility exceed the present evidence (10, 11).

### 4.8 Lifestyle-related environmental exposures

Tobacco smoke exposes users to oxidants, polycyclic aromatic hydrocarbons, and metals, and is a modifiable reproductive-health risk because it can increase oxidative stress and impair vascular function (26, 76). Heavy alcohol use can disrupt hypothalamic-pituitary-gonadal signalling, worsen liver disease, impair sexual function, and alter nutrition; sleep loss, sedentary behaviour, obesity, and recreational drug use often coexist with these exposures (77, 78). Lifestyle advice should not blame patients, because work conditions, housing, income, marketing, and mental health shape what changes are feasible (79, 80).

Physical activity has a dose-dependent relationship with reproductive health. Moderate regular exercise supports cardiometabolic function, whereas extreme training, energy deficiency, overheating, or anabolic-steroid use can create endocrine and testicular stress in susceptible men (11, 81).

### 4.9 Summary of environmental effects

Environmental evidence supports a precautionary model: reduce avoidable exposure, identify high-risk work and living conditions, and avoid individual causal claims from a single biomarker or semen result. Source control through regulation, safer work design, clean air, and surveillance is more reliable than relying on individual behavioural changes alone (10, 11).

## 5 Pharmacological influences on male reproductive health

Pharmacological agents and substances of misuse can affect male reproductive health through direct gonadotoxicity, suppression of hypothalamic-pituitary-gonadal signalling, hyperprolactinaemia, vascular effects, or impairment of erectile and ejaculatory function (75, 82). The magnitude and reversibility of these effects vary by drug, dose, duration, underlying disease, and baseline reproductive reserve; medicine-related concerns therefore require individualised review rather than abrupt treatment cessation (2, 59). **Table 3** summarises the principal pharmacological and substance-related influences, their major reproductive concerns, the interpretation of available evidence, and appropriate clinical responses.

**Table 3:** Pharmacological and substance-related influences: evidence, principal concern, and clinical response.

Exposure or treatment	Main reproductive concern	Evidence interpretation	Possible clinical response
Chemotherapy and testicular/pelvic radiation	Direct germ-cell depletion, azoospermia, endocrine injury	High-quality guideline support for early fertility-preservation counselling; risk varies by regimen, dose, field, and baseline reserve (59).	Discuss sperm cryopreservation before therapy; do not delay urgent cancer treatment without oncology coordination.
Exogenous testosterone and anabolic-androgenic steroids	Suppressed LH/FSH and low intratesticular testosterone; oligozoospermia/azoospermia	Well-established biological mechanism and consistent clinical literature (51, 83).	Avoid testosterone when fertility is desired unless managed by a specialist; disclose non-prescribed steroid use.
Antihypertensives	Erectile dysfunction or altered sexual performance in some users	Hypertension is often a major confounder; effects differ by class and patient. Nebivolol may be less adverse for erection in some settings (84).	Review cardiovascular risk and treatment; do not stop antihypertensives without clinician-led adjustment.
Antidepressants	Reduced libido, delayed ejaculation, orgasmic and erectile difficulties	A 2025 network meta-analysis included 30 RCTs and 18,157 participants; sexual adverse-effect risk differed across agents (35).	Assess symptoms directly; balance sexual effects against effective treatment of depression/anxiety; consider alternatives collaboratively.
Antipsychotics	Hyperprolactinaemia, low testosterone, sexual dysfunction	Drug-induced hyperprolactinaemia has variable presentation and requires clinical confirmation (85).	Check symptoms and endocrine status where indicated; coordinate with psychiatry rather than stopping treatment abruptly.
Long-term opioids	Opioid-induced androgen deficiency, low libido, erectile dysfunction	Endocrine/sexual effects are biologically plausible and clinically recognised; pain and comorbidity may contribute (86).	Review pain regimen, dependence, sleep, mood, and testosterone symptoms; use multidisciplinary care.
Cyclophosphamide and selected immunosuppressants	Potential gonadotoxicity; disease and drug effects may coexist	Risk is drug- and cumulative-dose-specific; active inflammatory disease can itself impair reproductive health (87).	Use specialist preconception counselling and consider sperm banking before high-risk exposure.
Alcohol, recreational substances, non-prescribed drugs	Endocrine, vascular, oxidative, behavioural, and sexual effects	Evidence is heterogeneous and commonly confounded by dose, tobacco, socioeconomic factors, and co-use.	Offer non-stigmatising substance-use support; encourage cessation or reduction before conception attempts.
5-alpha-reductase inhibitors and alpha-blockers	Libido, ejaculatory or erectile effects; possible semen concerns in selected men	Medication effects must be distinguished from symptoms of benign prostatic disease and baseline sexual health. Reviews identify a need for individualised assessment (82).	Ask directly about sexual and ejaculatory effects; do not stop treatment abruptly without a prescriber-led plan.
Antiseizure medicine: valproate	Hormonal and semen-parameter changes reported in clinical and preclinical studies	A 2024 meta-analysis found evidence of adverse reproductive associations, but clinical studies remain heterogeneous in design and indication (49).	For men planning conception, review seizure control, alternatives and reproductive monitoring with neurology; seizure stability remains essential.
Selective oestrogen receptor modulators and aromatase-directed therapy	Off-label attempts to improve endogenous gonadotropin/testosterone signalling in selected men	Meta-analyses report improvements in some hormonal and semen outcomes, but studies are heterogeneous and patient selection matters (42, 53, 88).	Use only after aetiological assessment and specialist counselling; avoid presenting empirical therapy as universally effective.
Anti-rheumatic drugs	Cyclophosphamide and sulfasalazine are prominent reproductive concerns; active disease may also impair fertility	Reviews support preconception discussion because risks vary by compound, cumulative dose and disease control (87, 89).	Coordinate rheumatology, reproductive medicine and fertility preservation before high-risk treatment.

A suspected drug-related reproductive problem should trigger review, not unsupervised discontinuation. The underlying disease may be the major cause of symptoms in some patients.

Note. A suspected drug-related reproductive problem should trigger review, not unsupervised discontinuation. The underlying disease may be the major cause of symptoms in some patients.

### 5.1 Overview of drug-induced reproductive effects

Medicines can protect fertility by controlling inflammation, infection, cancer, pain, seizures, diabetes, hypertension, or psychiatric illness. They may also affect fertility through direct gonadotoxicity, suppression of gonadotropins, altered testosterone or prolactin, sexual dysfunction, impaired ejaculation, or indirect effects on sleep, weight, mood, and vascular function (75, 82). The clinical question is therefore whether therapeutic benefit outweighs reproductive risk and whether safer alternatives, monitoring, or fertility preservation are warranted (1, 2).

A 2026 systematic review and meta-analysis of 41 randomized controlled trials involving 3,615 men with idiopathic male infertility found that antioxidant interventions were associated with higher sperm concentration (SMD 1.09, 95% CI 0.10-2.08), progressive motility (SMD 1.11, 95% CI 0.32-1.90), total motility (SMD 0.69, 95% CI 0.26-1.13), and normal morphology (SMD 0.43, 95% CI 0.05-0.82) (34). However, prediction intervals crossed the null and certainty was very low, so these estimates do not support indiscriminate empirical treatment (34).

The strength of evidence differs markedly across drug classes. Cancer therapy and exogenous testosterone have well-established reproductive implications, whereas many other suspected medication effects are drawn from animal studies, case reports, short trials, adverse-event data, or observational studies in which the underlying disease is a major confounder (75, 82).

### 5.2 Chemotherapeutic and anticancer drugs

Cancer can impair reproductive health before treatment through inflammation, fever, malnutrition, endocrine disturbance, testicular involvement, and psychological stress. Alkylating agents and relevant radiation fields can additionally deplete germ cells, while the probability of recovery depends on regimen, cumulative dose, age, and baseline reserve (59, 75).

The 2025 ASCO guideline recommends discussing fertility preservation as early as possible and promptly referring appropriate patients to reproductive specialists (59). Sperm cryopreservation before treatment is the established first-line option for postpubertal males who can provide an ejaculate, while specialist retrieval approaches may be considered when necessary (2, 59).

Fertility preservation preserves an option rather than guaranteeing future biological parenthood. Counselling should address expected treatment urgency, likely risk, time available for collection, use and storage arrangements, costs, and the fact that life-saving oncology treatment should not be delayed without coordinated specialist advice (1, 59).

### 5.3 Hormonal drugs and anabolic steroids

Exogenous testosterone is often incorrectly viewed as a fertility treatment because it can improve libido or symptoms in selected hypogonadal men. It usually suppresses hypothalamic and pituitary gonadotropins, lowers intratesticular testosterone, and can cause marked oligozoospermia or azoospermia despite a high serum testosterone concentration (51, 90). Anabolic-androgenic steroid misuse produces similar suppression and may also cause testicular atrophy, dyslipidaemia, hepatic injury, and mood disturbance (83, 91).

Recovery after discontinuation is variable and depends on duration, dose, compound, age, baseline fertility, and concurrent substances. Men who wish to conceive should disclose prescribed testosterone, anabolic agents, bodybuilding products, and other hormonal supplements to a reproductive urologist or endocrinologist rather than undertake unsupervised cessation or unregulated “post-cycle” treatment (51, 90).

### 5.4 Antihypertensive medications

Hypertension itself can cause erectile dysfunction through endothelial dysfunction, arterial stiffness, impaired penile blood flow, and shared metabolic risk (54, 84). Some antihypertensives may contribute to sexual effects in susceptible patients, but effects differ by class and individual and must be separated from the disease itself (82, 84).

A medication should not be stopped because of embarrassment or unverified internet advice. Uncontrolled hypertension raises cardiovascular risk and may worsen erectile function; clinician-led review can consider dose adjustment, a different class, or treatment of erectile dysfunction while protecting blood-pressure control (54, 84).

### 5.5 Antidepressants and antipsychotic drugs

Depression, anxiety, psychosis, and bipolar disorder can impair sexual function through reduced desire, anhedonia, disrupted sleep, low confidence, relationship strain, and autonomic arousal. Effective treatment can therefore improve reproductive wellbeing, even when some psychotropics have sexual adverse effects (35, 92). Selective serotonin reuptake inhibitors can cause reduced libido, delayed ejaculation, anorgasmia, or erectile difficulty in some users, with risk varying across agents (35, 75).

Antipsychotic medicines may affect sexual and reproductive function directly or through hyperprolactinaemia. Elevated prolactin can suppress gonadotropins, reduce testosterone, and contribute to low libido or erectile and ejaculatory dysfunction, although disease-related sexual dysfunction is also common (85, 93). Medication change should be coordinated with psychiatric care rather than undertaken abruptly (1, 85).

### 5.6 Opioids and analgesic drugs

Long-term opioid exposure can suppress the hypothalamic-pituitary-gonadal axis and contribute to low testosterone, reduced libido, erectile dysfunction, fatigue, low mood, and reduced bone health (75, 86). Pain, cancer, trauma, sleep disruption, and depression may also contribute, so reproductive counselling should be integrated with safe and effective pain management (2, 86).

Where opioid-induced androgen deficiency is suspected, clinicians should review analgesic indication, dose, duration, other sedatives, sleep apnoea risk, symptoms, and reproductive plans. Withdrawal is not an automatic solution; multimodal pain care, dependence treatment, and endocrine referral may be required (1, 86).

### 5.7 Anti-inflammatory and immunosuppressive agents

Anti-inflammatory and immunosuppressive therapies illustrate an important principle: the disease being treated may threaten fertility more than the drug. Severe autoimmune disease, chronic inflammation, vasculitis, and organ transplantation can disturb endocrine function, sexual health, and spermatogenesis through cytokine activity, organ dysfunction, fever, and poor nutrition (87, 89). Cyclophosphamide is a recognised gonadotoxic concern, whereas other agents require compound-specific counselling rather than blanket statements (75, 87).

Preconception counselling should cover disease activity, cumulative drug exposure, alternative treatments, sperm banking before a high-risk course, and the safety considerations relevant to a prospective partner and future pregnancy. Broad claims that all immunosuppression causes male infertility are inaccurate and can lead to harmful treatment withdrawal (87, 89).

### 5.8 Antimicrobial and antiviral drugs

Infection itself can impair fertility through fever, orchitis, epididymitis, obstruction, inflammation, and altered seminal conditions. Antimicrobial treatment frequently protects fertility by preventing or treating these complications, and medication effects must be distinguished from effects of the infection itself (75, 94). For HIV, hepatitis, tuberculosis, and recurrent genital infection, reproductive planning should be coordinated with infectious-disease care rather than based on unsupervised treatment interruption (1, 94).

Antiretroviral therapy has transformed health and reproductive options for people living with HIV. Counselling should prioritise viral suppression, transmission prevention, sexual function, and safe conception rather than suggesting that effective treatment should be withheld because of speculative fertility concerns (1, 94).

Available human evidence does not support a simple separation in which semen changes are attributed exclusively either to HIV itself or to antiretroviral therapy. Chronic viral infection, immune activation, comorbidity, fever, lifestyle factors, and treatment exposure can coexist, and much of the published semen literature reflects older or mixed regimens. Evidence for independent semen toxicity of specific contemporary antiretroviral classes, particularly current integrase-inhibitor-based regimens, remains insufficient for a class-wide causal conclusion. The clinically appropriate response is therefore to maintain effective viral suppression, investigate other causes of abnormal semen parameters, and seek infectious-disease or reproductive specialist input if a treatment-related effect is suspected rather than interrupting ART (1, 94).

### 5.9 Drugs of abuse

Cannabis, cocaine, methamphetamine, alcohol, non-prescribed opioids, anabolic agents, and inhalants can affect reproduction through endocrine disruption, oxidative stress, vascular injury, risky sexual behaviour, poor nutrition, sleep loss, and psychiatric symptoms (78, 79). A non-judgemental substance history is more useful than a moral warning because it enables treatment of dependence, infection prevention, assessment of sexual dysfunction, and discussion of fertility goals (1, 80).

Recovery after stopping substances may take time because new spermatogenic output is not reflected immediately in semen. Fertility counselling can therefore use reproductive goals as a non-stigmatising opportunity for recovery, while still investigating other medical or structural causes of infertility (6, 79).

### 5.10 Fertility preservation and pharmacological counselling

Fertility preservation is most effective when introduced before gonadotoxic therapy or irreversible surgery. It requires timely discussion of reproductive goals, explanation of risk, referral, and practical support with consent, cost, transport, and scheduling (2, 59). Sperm cryopreservation is established for postpubertal males, while prepubertal testicular tissue cryopreservation remains investigational in many settings (1, 59).

## 6 Disease influences on male reproductive health

Disease-related impairment of male reproductive health may arise from direct testicular or reproductive-tract injury, endocrine disturbance, inflammation, oxidative stress, vascular dysfunction, genetic abnormalities, or impaired sexual and ejaculatory function. Infectious, metabolic, cardiovascular, renal, hepatic, neurological, autoimmune, and malignant conditions may therefore affect fertility through distinct but overlapping pathways, and the likely mechanism should guide clinical assessment rather than assumptions based on diagnosis alone (2, 9). **Table 4** summarises the principal disease conditions associated with male reproductive impairment, their proposed mechanisms, evidence anchors, and priority clinical responses.

**Table 4:** Disease conditions, pathways to reproductive impairment, evidence anchors, and clinical priorities.

Condition	Principal reproductive pathway	Evidence	Clinical priority
Diabetes mellitus	Hyperglycaemia, neuropathy, endothelial dysfunction, oxidative stress, endocrine alteration	Systematic review/meta-analysis identifies several adverse fertility-related outcomes in type 1 diabetes, with heterogeneous methods (41).	Optimise glycaemia and cardiovascular risk; assess erection, ejaculation, testosterone symptoms, and semen where appropriate.
Obesity/metabolic syndrome	Inflammation, aromatisation, insulin resistance, raised scrotal temperature, sleep apnoea	BMI meta-analysis found poorer semen outcomes across heterogeneous studies (30). Weight-loss meta-analysis: 12 studies, 345 men, improved concentration, motility, and DNA fragmentation (31).	Support sustainable weight management while investigating other reversible causes.
Varicocele	Thermal stress, venous stasis, oxidative stress, altered testicular perfusion	A 2024 meta-analysis synthesised 351 studies and reported semen-parameter improvements, but with high heterogeneity; RCT pregnancy evidence remains less certain (32, 33).	Consider repair when the varicocele is clinical/palpable, semen parameters are abnormal, infertility is otherwise unexplained, and the female partner has good ovarian reserve; do not treat subclinical varicocele or normal semen solely for fertility (9).
Infections	Orchitis, epididymitis, fever, inflammation, obstruction, altered seminal environment	Viral and genital infections can affect reproductive function, but presence of infection markers in semen alone does not prove causality (94).	Test and treat promptly, vaccinate where recommended, manage acute scrotal symptoms urgently, include partner care.
Cardiovascular disease	Endothelial dysfunction and reduced penile blood flow	Princeton IV recommends cardiovascular risk assessment in men with erectile dysfunction (54).	Screen and manage hypertension, diabetes, lipids, smoking, sleep, and medication effects.
Genetic/chromosomal disorders	Impaired spermatogenesis or obstructive azoospermia	EAU: AZFc deletions account for about 65–70% of clinically relevant Y microdeletions; testicular sperm may be retrieved in 50–75% of AZFc cases (European Association of Urology [EAU], 2026).	Offer indication-based karyotype, Y-microdeletion, and CFTR evaluation with genetic counselling.
Neurological and psychological conditions	Autonomic dysfunction, altered ejaculation, depressive symptoms, medication effects, relationship strain	Depression and ED are bidirectionally associated; sexual dysfunction is common in schizophrenia (93, 95).	Provide sexual-health screening, mental-health support, rehabilitation, and assisted-ejaculation/retrieval options where needed.
Chronic kidney/liver disease	Hormonal disruption, uraemia or altered metabolism, vascular disease, fatigue, medication effects	Kidney disease review highlights reproductive consequences and need for life-course counselling (96).	Address disease control, endocrine symptoms, transplant medicines, and early reproductive planning.
Cancer	Disease burden plus surgery, chemotherapy, radiotherapy, endocrine therapy	ASCO recommends timely fertility-preservation discussion and referral before treatment (59).	Offer sperm cryopreservation and survivorship follow-up for fertility and sexual health.
Chronic autoimmune and inflammatory disease	Inflammation, organ dysfunction, sexual symptoms, and drug effects can coexist	Reproductive effects must be attributed cautiously because disease activity and medication exposure are intertwined (87, 89).	Control disease activity and offer preconception counselling before altering effective therapy.
Mumps or other viral orchitis; HIV/HPV-related illness	Testicular inflammation, fever, immune activation, sexual or reproductive-tract effects	Viral associations vary by pathogen and study design. Reviews support prompt evaluation rather than inference from semen findings alone (94, 97).	Use vaccination where indicated, timely testing/treatment, partner care, and fertility counselling after clinically significant orchitis.
Cryptorchidism, testicular torsion or traumatic testicular injury	Impaired germ-cell development, ischaemia, scarring, endocrine loss or obstructive consequences	Guidelines recognise these as important historical factors in male-infertility assessment, with prognosis depending on timing and severity (2, 9).	Urgent acute scrotal assessment; early specialist follow-up and fertility evaluation when relevant.
Genitourinary infection in Nigerian and other resource-constrained settings	Inflammation, epididymal obstruction, altered seminal environment, delayed treatment	Nigerian and African reviews identify infections and environmental risks as important contextual concerns, while emphasising that much regional evidence is observational and requires updated population-based studies (14, 36).	Strengthen STI prevention, accessible diagnosis, antimicrobial stewardship, partner management and environmental-health protection.

A disease association does not imply that every affected man will experience infertility. Clinical examination and couple-centred assessment are required to identify the relevant pathway in an individual patient.

### 6.1 Infectious diseases

Infections can impair male reproductive health through orchitis, epididymitis, prostatitis, fever, inflammation, obstruction, oxidative stress, or changes in seminal plasma (6, 94). Sexually transmitted infections such as chlamydia and gonorrhoea can damage the reproductive tract when untreated, while mumps orchitis remains a recognised cause of testicular inflammation and sometimes atrophy (1, 94).

Recent pooled evidence adds pathogen-specific detail. A 2026 systematic review identified 2,720 records, retained 51 studies for qualitative synthesis, and included 35 in quantitative analyses (39). Male genital-tract infections were associated with impairment of selected semen parameters, particularly in infertile populations; HPV was associated with increased antisperm-antibody prevalence (OR 10.63, 95% CI 1.49–75.93), and hepatitis B infection with miscarriage risk (OR 1.43, 95% CI 1.05–1.93), while many pregnancy, live-birth and ART outcomes were nonsignificant and heterogeneous (39).

The presence of leucocytes or microbial material in semen should not automatically be equated with infertility or justify repeated antibiotics. Testing, treatment, partner management, and follow-up should be guided by symptoms, examination, sexual history, validated tests, and local antimicrobial-resistance guidance (1, 94).

### 6.2 Endocrine and metabolic disorders

Diabetes mellitus can affect erection, ejaculation, libido, testosterone, and sperm function. Hyperglycaemia promotes oxidative stress and advanced glycation, while endothelial dysfunction and autonomic neuropathy can impair erection and ejaculation (41, 52). A systematic review and meta-analysis found several adverse fertility-related outcomes in type 1 diabetes, but heterogeneity across methods and endpoints means that diabetes is a risk context rather than a universal infertility diagnosis (1, 41).

The practical response is proactive assessment rather than deterministic counselling. Optimising glycaemic control, vascular risk, weight, sleep, medication, and mental health can improve general wellbeing and may address important pathways to erectile or ejaculatory dysfunction (1, 52).

Obesity adds inflammation, insulin resistance, sleep-disordered breathing, raised scrotal temperature, altered hormonal feedback, and psychological strain (30, 43). A 2024 meta-analysis associated higher body mass index with poorer semen measures, while a 2025 meta-analysis of 12 studies involving 345 men found improvements in concentration, progressive motility, and DNA fragmentation after weight-loss interventions (30, 31).

A 2026 systematic review and meta-analysis specifically evaluated reproductive outcomes after obesity interventions, including lifestyle measures, pharmacotherapy, and bariatric approaches (98). The evidence supports the biological relevance of weight reduction but remains heterogeneous across intervention type, degree of weight loss, semen endpoints, and fertility outcomes; counselling should therefore focus on sustainable cardiometabolic improvement rather than a guaranteed fertility effect (98).

Weight-management interventions should be framed as long-term health support rather than a requirement imposed before fertility care. Nutritional assessment, sleep-apnoea evaluation, physical activity planning, medication review, and mental-health support can be matched to the person’s circumstances and reproductive timeline (1, 31).

Other endocrine disorders include primary and secondary hypogonadism, hyperprolactinaemia, thyroid dysfunction, Cushing syndrome, congenital adrenal disorders, and pituitary disease. They may present with low libido, gynecomastia, erectile dysfunction, small testes, infertility, or abnormal semen parameters, and should be treated according to confirmed aetiology rather than empirical hormone use (2, 9).

### 6.3 Cardiovascular diseases

Hypertension, dyslipidaemia, diabetes, atherosclerosis, and coronary artery disease can impair sexual function through endothelial dysfunction and reduced penile arterial inflow (54, 84). Erectile dysfunction may precede recognised cardiovascular disease, so it should prompt assessment of blood pressure, glycaemia, lipids, body weight, smoking, sleep, mood, and medication effects (1, 54).

Treatment of cardiovascular risk can support both life expectancy and sexual health. Phosphodiesterase-5 inhibitors may be appropriate for selected patients, but they do not replace assessment of cardiovascular disease, medication interactions, or nitrate use (9, 54).

### 6.4 Testicular and reproductive tract disorders

Varicocele is a dilatation of the pampiniform plexus and a common potentially correctable finding in male infertility. Proposed mechanisms include increased scrotal temperature, impaired venous drainage, oxidative stress, altered intratesticular perfusion, reflux of metabolites, and endocrine disruption (9, 32). A varicocele is not automatically harmful, so management depends on palpability, semen findings, symptoms, testicular size, couple factors, and reproductive timing (2, 9).

Other testicular and tract disorders require equally mechanism-specific management. Cryptorchidism, torsion, trauma, epididymo-orchitis, obstruction, congenital absence of the vas deferens, and testicular cancer can affect germ-cell development, sperm transport, or endocrine reserve; acute scrotal pain must always be evaluated urgently (2, 9).

Cannarella et al. (32) analysed 351 studies and reported improvements in conventional semen parameters after varicocele repair in pooled before-after data, while also identifying high heterogeneity and publication bias for some outcomes. A separate meta-analysis of four randomised trials involving 349 participants did not show a statistically clear improvement in spontaneous pregnancy after primary varicocele surgery (33). These findings support shared decision-making rather than guaranteed outcomes (9, 32).

A complementary 2026 meta-analysis screened 1,622 records and included 32 studies. Men with varicocele had higher FSH (MD 2.18 IU/L, 95% CI 1.37–2.99) and LH (MD 0.63 IU/L, 95% CI 0.08–1.17) and lower total testosterone (MD −0.71 ng/mL, 95% CI −1.20 to −0.23) than men without varicocele; free testosterone, prolactin, and estradiol did not differ significantly (38). These findings support possible endocrine as well as spermatogenic effects while not, by themselves, determining surgical candidacy.

Current EAU guidance recommends treatment for infertile men with a clinical (palpable) varicocele, abnormal semen parameters, and otherwise unexplained infertility when the female partner has good ovarian reserve. In contrast, repair is not recommended for a subclinical varicocele or for infertile men with normal semen analysis. These criteria should be integrated with symptoms, testicular findings, the female partner’s age and ovarian reserve, and the couple’s reproductive timeline, because the expected value of surgery declines when a time-sensitive assisted-reproduction pathway is more appropriate (9).

Cryptorchidism, testicular torsion, trauma, epididymo-orchitis, obstruction, hydrocele, testicular cancer, and congenital absence of the vas deferens can compromise reproduction through different mechanisms. Torsion is an emergency because testicular salvage depends on time (1, 2). Testicular cancer requires urgent oncological care, with fertility preservation considered before orchiectomy, chemotherapy, or radiotherapy where feasible. Congenital bilateral absence of the vas deferens is commonly associated with cystic fibrosis transmembrane conductance regulator variants and often requires genetic counselling and assisted reproduction (1, 2). Anatomy should guide testing; not all male infertility is diffuse “poor sperm quality.”

### 6.5 Autoimmune and inflammatory conditions

Autoimmune and chronic inflammatory diseases may reduce male reproductive health through cytokine activity, fever, vascular injury, fatigue, pain, depression, renal or hepatic involvement, and treatment effects (87, 89). Disease activity matters because uncontrolled inflammation can reduce sexual function and fertility, whereas effective therapy may restore broader wellbeing (1, 87).

Rarely, autoimmune orchitis may directly affect testicular tissue, but in most patients the interaction is multifactorial rather than a single lesion. This reinforces the need to interpret semen and hormone findings alongside disease activity, organ function, and treatment exposure (87, 89).

### 6.6 Genetic and chromosomal disorders

Genetic factors are important in severe oligozoospermia, azoospermia, congenital absence of the vas deferens, disorders of sex development, and some cases of recurrent pregnancy loss (9, 99). Klinefelter syndrome can cause primary testicular failure, while Y-chromosome microdeletions affect regions required for spermatogenesis; AZFc deletions have a more favourable sperm-retrieval prognosis than complete AZFa or AZFb deletions (9, 100).

Genetic findings can have implications for assisted reproduction, offspring, family members, renal anomalies, and psychological wellbeing. They should be explained by clinicians able to discuss inheritance, uncertainty, and reproductive options without implying that a genetic result determines a person’s value or future family life (1, 99).

Genetic testing is not a screening test for every man. It is indicated by semen profile, examination, endocrine findings, family history, and suspected obstruction, and should be accompanied by counselling on inheritance, reproductive options, and uncertainty (2, 99).

### 6.7 Neurological and psychological conditions

Neurological conditions may impair erection, ejaculation, genital sensation, and the ability to produce a semen sample. Spinal cord injury, multiple sclerosis, diabetes-related neuropathy, pelvic surgery, stroke, and neurodegenerative disease can alter autonomic and somatic pathways (2, 9). Assisted ejaculation, electroejaculation, sperm retrieval, and assisted reproduction can help selected patients, but require diagnosis-specific and couple-centred planning (1, 9).

Psychological conditions matter directly. Depression and erectile dysfunction have a bidirectional relationship, while schizophrenia is associated with a high burden of sexual dysfunction through illness, medication, physical comorbidity, and social exclusion (93, 95). Screening, couples counselling where desired, and mental-health referral are legitimate elements of fertility care (1, 2).

### 6.8 Chronic kidney and liver disease

Chronic kidney disease can disrupt reproductive health through uraemia, altered hypothalamic-pituitary-gonadal signalling, hyperprolactinaemia, anaemia, fatigue, vascular disease, inflammation, and medication burden. Chronic liver disease can likewise reduce testosterone through altered steroid metabolism, malnutrition, alcohol-associated harm, and systemic illness; early reproductive counselling is therefore appropriate in both conditions (96, 101).

Fertility and sexual-health conversations should begin early in kidney and liver disease rather than only after advanced organ failure or transplantation. Earlier discussion allows patients to consider medication effects, dialysis or transplant timing, sperm banking, and the role of their wider health priorities (96, 101).

Chronic liver disease can alter sex-hormone metabolism, increase oestrogenic effects, reduce testosterone, and worsen nutritional and vascular health. Alcohol-related liver disease adds direct alcohol exposure and psychosocial burden (1, 2). Advanced respiratory, gastrointestinal, endocrine, and systemic inflammatory disease may produce similar effects through chronic illness, hypoxia, catabolism, fever, or treatment toxicity. Fertility care should not be delayed until disease is “perfectly controlled”; reproductive goals should be discussed early, while recognising that stabilising the underlying condition may be the most effective reproductive intervention (1, 2).

### 6.9 Cancer and male reproductive health

Cancer affects male reproduction through both disease and treatment. Testicular cancer can alter semen quality before orchiectomy, while pituitary tumours, prostate cancer treatment, lymphoma, and leukaemia can affect endocrine function, ejaculation, erections, or spermatogenesis through disease burden and therapy (59, 75). Fertility preservation should be offered as part of cancer care rather than reserved for men who are already partnered or currently planning pregnancy (1, 59).

## 7 Mechanisms linking environmental, pharmacological, and disease factors to reproductive dysfunction

Environmental toxicants, therapeutic medicines, substance use, and disease conditions may impair male reproductive health through interconnected biological mechanisms rather than through isolated pathways. Oxidative stress, endocrine disruption, inflammation, sperm DNA damage, vascular impairment, and neurological dysfunction can jointly affect spermatogenesis, testosterone production, sperm maturation, erectile function, ejaculation, and fertility outcomes (9, 10). **Figure 2** summarises these converging pathways and illustrates how diverse exposures and disease states can lead to reduced semen quality, hormonal imbalance, sexual dysfunction, and male infertility.

**Figure 2:**
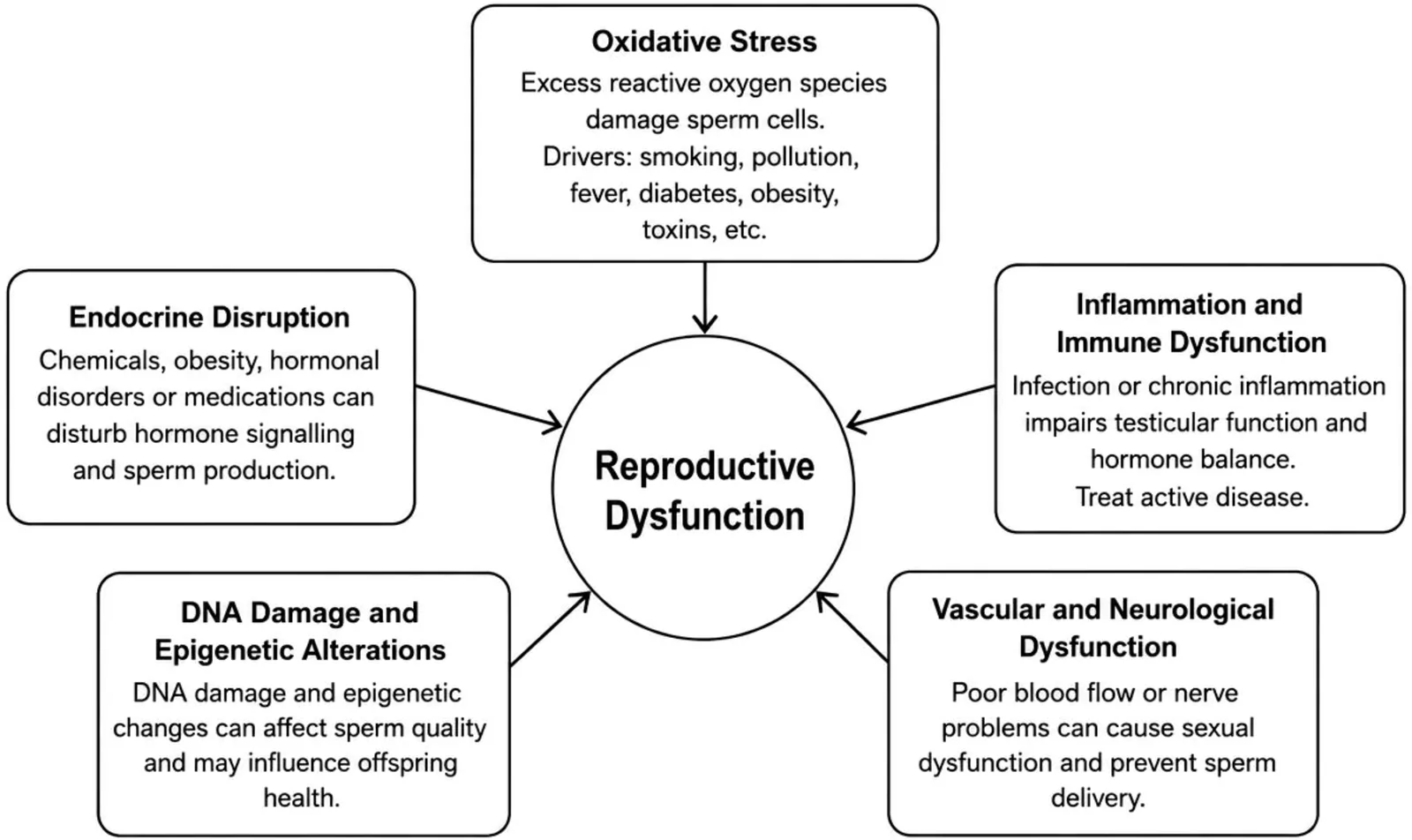
Conceptual pathways linking environmental exposures, pharmacological agents, and disease conditions to male reproductive dysfunction.

### 7.1 Oxidative stress

Oxidative stress occurs when reactive oxygen species exceed antioxidant defences. Small amounts contribute to normal sperm signalling, but excessive levels can damage sperm lipids, proteins, mitochondria, and DNA because sperm membranes are rich in polyunsaturated fatty acids and repair capacity is limited(46). Tobacco, pollution, pesticides, metals, fever, varicocele, diabetes, obesity, infection, and chronic inflammation can all contribute to this pathway (10, 68).

Oxidative stress is a convergent mechanism, not a complete diagnosis. Clinical care should first identify the driver, such as smoking, varicocele, fever, diabetes, infection, obesity, or workplace exposure, before considering any adjunctive therapy (9, 46).

### 7.2 Endocrine disruption

Endocrine disruption may arise from environmental chemicals, obesity-related aromatisation, pituitary disease, hyperprolactinaemia, chronic opioid exposure, exogenous testosterone, anabolic steroids, and severe systemic illness (51, 55). The shared outcome is altered hypothalamic-pituitary-gonadal signalling, although the meaning of a testosterone value depends on symptoms, gonadotropins, sex hormone-binding globulin, testicular volume, and fertility goals (2, 9).

### 7.3 Inflammation and immune dysfunction

Inflammation can affect the testes, epididymis, prostate, vasculature, and neuroendocrine system. Acute infection may cause fever and transient spermatogenic impairment, while chronic inflammatory disease can sustain cytokine exposure, oxidative injury, and endocrine disturbance (87, 94). The aim is to identify and treat active disease rather than diagnose “silent infection” without evidence and expose patients to unnecessary antibiotics (1, 9).

The blood-testis barrier ordinarily limits immune recognition of developing germ cells. Trauma, infection, torsion, or chronic inflammation may disrupt this environment, but the variable clinical role of antisperm antibodies means that testing is not routinely indicated for every man with infertility (2, 9).

### 7.4 DNA damage and epigenetic alterations

Sperm DNA integrity is influenced by oxidative stress, defective chromatin packaging, apoptosis, varicocele, fever, ageing, smoking, toxicant exposure, and genital-tract inflammation. During spermiogenesis, most histones are replaced by transition proteins and protamines to achieve highly compact chromatin; incomplete protamination or abnormal retention of histones at developmentally important loci can leave DNA more vulnerable to damage and may alter regulatory information delivered by the sperm. DNA fragmentation assays capture aspects of strand damage, whereas epigenetic assays interrogate different biological layers and should not be treated as interchangeable clinical tests (46, 102).

Epigenetic information in sperm includes DNA methylation, retained histone modifications, and small non-coding RNAs. Experimental and human observational research indicates that obesity, smoking, diet, endocrine-disrupting exposures, and other paternal conditions can be associated with changes in these marks. Small RNAs, including microRNAs and tRNA-derived fragments, can also be remodelled during epididymal maturation through extracellular-vesicle-mediated transfer. Animal exposure models provide important mechanistic evidence that altered DNA methylation, histone-retention patterns, and sperm RNAs can persist across generations; however, direct demonstration of stable transgenerational inheritance and clinically predictable offspring effects in humans remains limited. Accordingly, mechanistic plausibility should be distinguished from established human causal inheritance (10, 102).

Recent reviews further support the mechanistic breadth of this field (103). Human male-infertility literature now describes DNA methylation, histone modifications, chromatin remodelling, and coding and non-coding sperm RNAs as interacting layers of epigenetic regulation rather than isolated biomarkers (104–106). These sources reinforce the need to distinguish association and mechanistic plausibility from clinically validated prediction of fertility or offspring outcomes.

Emerging biomarker research is evaluating sperm DNA methylation signatures, chromatin/protamine measures, microRNA and other small-RNA profiles, extracellular vesicles, metabolomic signatures, and sperm or seminal-plasma proteomics. These approaches may eventually improve phenotyping of otherwise unexplained infertility or identify exposure-related pathways, but analytical platforms, reference ranges, reproducibility, and prospective links with pregnancy or live birth are not yet sufficiently standardised for routine population screening. At present, these biomarkers are most appropriately considered research tools or selectively used specialist tests rather than deterministic predictors of fertility or offspring health (9, 102).

### 7.5 Vascular and neurological dysfunction

Vascular and neurological dysfunction are central to sexual function. Diabetes, hypertension, dyslipidaemia, smoking, obesity, kidney disease, pelvic surgery, and some medicines can reduce penile blood flow or neural signalling (54, 96). Erectile dysfunction, retrograde ejaculation, and anejaculation may prevent sperm deposition even when spermatogenesis is adequate, making direct sexual-history questions essential (2, 9).

A reproductive evaluation that ignores sexual function is incomplete. Direct, respectful questions about desire, erection, ejaculation, orgasm, pain, intercourse frequency, and reproductive timing can reveal treatable barriers that a semen analysis alone will miss (1, 2).

## 8 Assessment and diagnosis of male reproductive health problems

Assessment of male reproductive health requires a structured and couple-centred approach because infertility, sexual dysfunction, hormonal disturbance, obstruction, and exposure-related injury may occur separately or together (2, 9). **Figure 3** summarises the diagnostic pathway, beginning with clinical and exposure history, physical examination, and semen analysis, followed by targeted hormonal, imaging, genetic, microbiological, and functional investigations where indicated. The pathway supports identification of reversible causes, timely referral for specialised care, and treatment decisions that reflect both the man’s clinical findings and the reproductive circumstances of the couple (2, 50).

**Figure 3:**
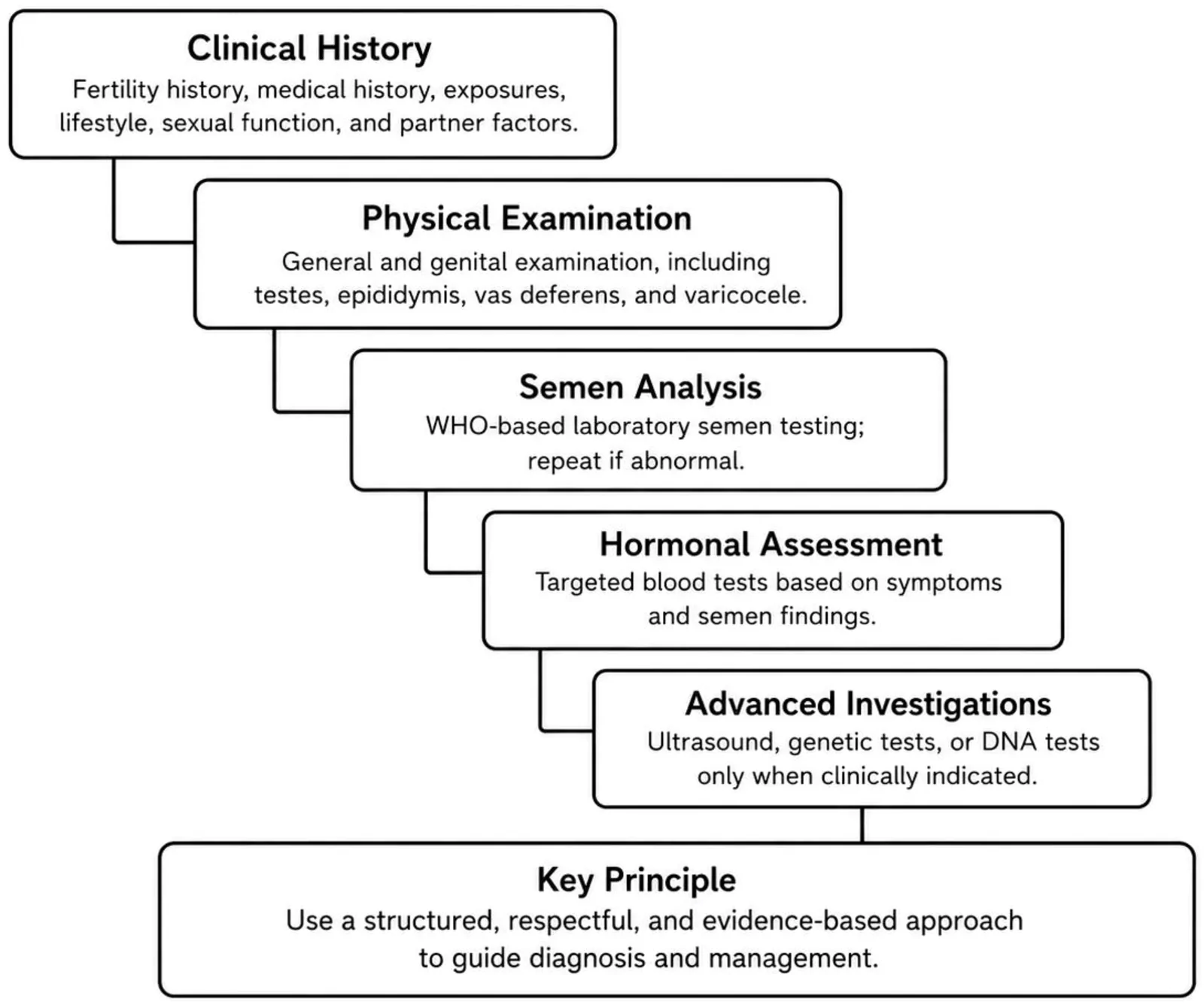
Stepwise clinical pathway for assessment and diagnosis of male reproductive health problems.

### 8.1 Clinical history

A detailed history is the foundation of male reproductive assessment. It should cover time attempting conception, intercourse and timing, prior conceptions or losses, puberty, cryptorchidism, torsion, trauma, mumps orchitis, genital surgery, infection, cancer, chronic disease, and family history (8; 9). Specific questions about work tasks, pesticides, metals, heat, radiation, smoking, alcohol, recreational substances, testosterone, supplements, and medicines are more useful than asking only whether a patient is “healthy” (2, 10).

History-taking should be non-judgemental and trauma-informed, particularly when asking about sexual activity, substance use, occupational hazards, violence, or mental health. A respectful conversation improves disclosure and helps distinguish exposure-related concern from symptoms that require urgent endocrine, urological, infectious-disease, or psychological assessment (1, 2).

The history should also include libido, erection, ejaculation, orgasm, pain, relationship stress, depression, anxiety, sleep, diet, activity, and financial barriers to care (1, 2). Parallel assessment of the female partner is essential because her age, ovarian reserve, tubal status, reproductive history, and timing can change the most appropriate next step (1, 9).

Couple-centred management requires simultaneous rather than sequential interpretation of male and female factors. Azoospermia or severe oligozoospermia should accelerate specialist male evaluation because endocrine, genetic, obstructive, or testicular causes may change the available treatment pathway and may require sperm retrieval or assisted reproduction. At the same time, the female partner’s age and ovarian reserve influence how much time can reasonably be allocated to repeated observation or corrective male treatment before proceeding to assisted reproduction. EAU guidance notes that age and ovarian reserve should inform timing and the choice between interventions such as male surgery and ART, and that earlier evaluation may be appropriate when the female partner is older than 35 years (1, 9).

The 2026 NICE fertility guideline provides an additional independent clinical framework. It recommends prompt repeat testing when azoospermia or severe oligozoospermia is detected, physical examination of the scrotum and testes after repeated abnormal semen analyses, and consideration of serum testosterone and gonadotrophins. For male-factor treatment, gonadotrophins are recommended for hypogonadotropic hypogonadism, whereas androgens should not be offered to treat semen abnormalities (107).

Where ovarian reserve is good and no major time-sensitive female factor is present, treatment of an appropriate reversible male condition may be considered before ART; the EAU varicocele recommendation is one example. Conversely, reduced ovarian reserve, advanced female reproductive age, or a male finding that is unlikely to recover within a useful timeframe may justify earlier coordinated ART referral. These decisions should integrate prognosis, patient preferences, treatment burden, cost, and the possibility of fertility preservation rather than applying semen thresholds in isolation (1, 9).

### 8.2 Physical examination

Physical examination should include body habitus, secondary sexual characteristics, gynecomastia, blood pressure, signs of endocrine disease, and abdominal or inguinal scars (2, 9). Genital examination assesses penile anatomy, testicular size and consistency, epididymal fullness, the vas deferens, varicocele, hydrocele, and masses, and should be private, explained, and chaperoned according to policy (1, 9).

### 8.3 Semen analysis

Semen analysis should be performed in a laboratory using WHO-based procedures. Collection conditions, abstinence period, sample handling, volume, concentration, total count, motility, morphology, vitality, and inflammation markers should be documented, and abnormal findings usually require repeat testing because biological and analytical variation is substantial (6, 50). Very low volume, azoospermia, or severe oligozoospermia can direct specific diagnostic pathways but do not define masculinity or predict fertility alone (8; 4).

### 8.4 Hormonal assessment

Hormonal assessment is targeted rather than universal. Morning total testosterone, follicle-stimulating hormone, luteinising hormone, prolactin, thyroid tests, and estradiol may be appropriate with azoospermia, severe oligozoospermia, low libido, erectile dysfunction, gynecomastia, small testes, or suspected pituitary disease (8; 2). Repeat testing and specialist interpretation are necessary when acute illness, obesity, medication, or altered sex hormone-binding globulin complicate the result (2, 9).

### 8.5 Advanced investigations

Advanced testing is selected according to the likely mechanism. Scrotal ultrasound and Doppler studies can clarify testicular lesions, volume, and uncertain venous anatomy, while post-ejaculatory urine testing, genetic testing, sperm DNA fragmentation testing, and testicular biopsy are used selectively (2, 9). Such tests should answer a clinical question and change management rather than be offered as unselective add-ons (1, 4).

Genetic testing is particularly relevant in azoospermia, severe oligozoospermia, suspected congenital absence of the vas deferens, and selected recurrent-loss contexts. Its value lies in clarifying prognosis and counselling, not in indiscriminate testing that may generate uncertain findings without a management plan (9, 99).

## 9 Prevention and management strategies

Prevention and management of male reproductive-health problems require an integrated approach that addresses environmental exposures, harmful substance use, medication-related risks, underlying diseases, sexual dysfunction, and barriers to timely fertility care (2, 9). Effective care combines early risk identification, treatment of reversible conditions, fertility preservation before gonadotoxic therapy, healthy behavioural support, and couple-centred counselling that reflects both partners’ reproductive circumstances (12, 59). **Figure 4** presents this integrated prevention-and-management pathway, showing how individual clinical care, workplace protection, environmental regulation, and public-health action can jointly protect male reproductive potential.

**Figure 4:**
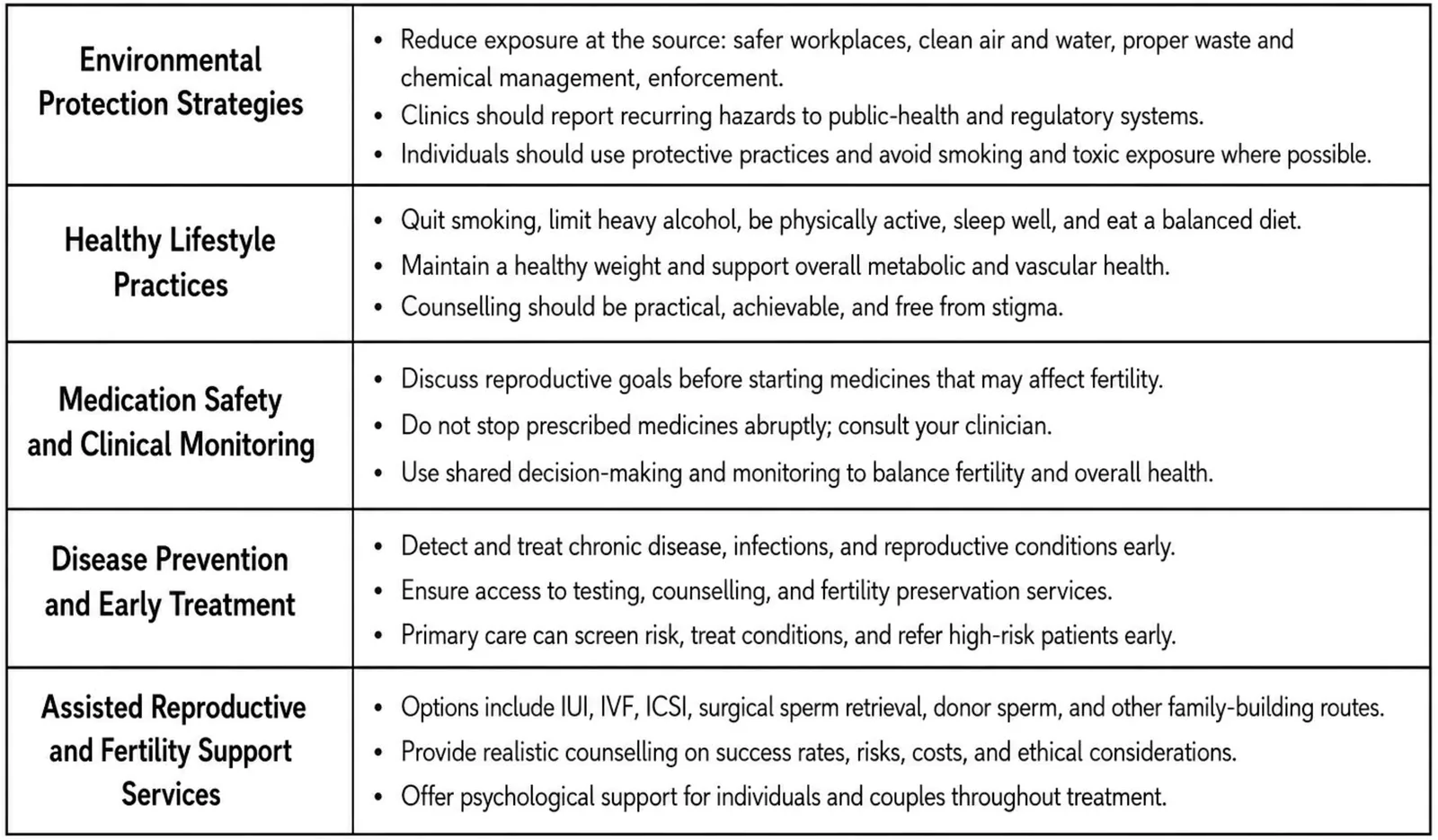
Integrated prevention and management pathway for protecting male reproductive health.

### 9.1 Environmental protection strategies

The most effective environmental prevention occurs upstream. Employers and regulators should minimise hazardous exposure through substitution, engineering controls, safe storage, training, appropriate personal protective equipment, hygiene facilities, monitoring, and enforcement (1, 72). Environmental protection requires action on air quality, contaminated water, industrial emissions, e-waste, pesticides, and informal work rather than advice that places the entire burden on individuals (10, 68).

Prevention is strengthened when clinics document occupational and environmental concerns and communicate recurring hazards to public-health and regulatory systems. This linkage recognises that a clinician can advise a patient, but cleaner production, enforcement, and safe work design are necessary to reduce exposure across populations (1, 10).

Individual measures still matter. Men with relevant occupational exposure can discuss job tasks, protective practice, heat, and conception plans with clinicians or occupational-health services, and should avoid smoking and unnecessary toxic exposure where feasible (2, 11).

### 9.2 Healthy lifestyle practices

A healthy lifestyle supports male reproduction through multiple pathways. Smoking cessation, lower heavy alcohol intake, regular physical activity, adequate sleep, balanced nutrition, and sustainable weight management can improve vascular, endocrine, and metabolic conditions that influence sexual and reproductive function (79, 81). Lifestyle interventions should be specific, feasible, and non-stigmatising, particularly where work, food environments, mental health, or financial stress constrain choice (1, 80).

Dietary counselling should emphasise sustainable dietary patterns and adequate nutritional status rather than unvalidated fertility-specific foods or supplement regimens. Evidence supports addressing overall cardiometabolic health, while evidence for many marketed antioxidant combinations remains variable and cannot replace aetiological evaluation (9, 43).

Lifestyle counselling works best when it is specific, feasible, and non-stigmatising. A man working two jobs may have limited time for exercise; one living near industry may be unable to control ambient air quality; a patient with depression may struggle with sleep or smoking cessation (1, 2). Clinical teams should identify barriers and use practical goals, referral to mental-health or addiction services, dietetic support, and occupational advocacy. Fertility-focused motivation can be valuable, but health improvements should not be made conditional on achieving pregnancy (1, 2).

### 9.3 Medication safety and clinical monitoring

Medication safety begins with a reproductive-goal conversation before treatment starts. Cancer patients should be offered fertility preservation before gonadotoxic therapy, and men considering testosterone, anabolic steroids, chronic opioids, selected immunosuppressants, or medicines causing marked sexual effects should be counselled about their reproductive plans (51, 59). Prescribed medicines should not be stopped abruptly because disease relapse, uncontrolled hypertension, seizure recurrence, psychiatric destabilisation, or severe pain can also harm health and sexual function (1, 82).

The central safety rule is that prescribed medicine should not be stopped abruptly because of fertility fears. Sudden discontinuation of antidepressants, antipsychotics, blood-pressure medicines, anticonvulsants, steroids, antiretrovirals, or pain therapy can cause relapse, withdrawal, hypertension, seizure, uncontrolled infection, or severe pain (1, 2). Shared decision-making protects both fertility and health. It should include realistic discussion of the strength of evidence, the potential reversibility of effects, and the role of monitoring or referral (1, 2).

### 9.4 Disease prevention and early treatment

Early diagnosis and treatment of diabetes, hypertension, obesity, sexually transmitted infections, varicocele, testicular disorders, endocrine disease, and cancer can prevent avoidable reproductive harm (1, 2). Access to semen assessment, endocrine testing, ultrasound, genetic counselling, fertility preservation, sexual-health care, and mental-health services is therefore an essential component of comprehensive prevention and care (1, 13).

Prevention also requires access. Semen analysis, endocrine testing, ultrasound, genetic counselling, fertility preservation, and sexual-health services are unavailable or unaffordable in many settings (1, 2). Primary-care clinicians can still make a substantial difference by taking a reproductive history, advising against non-prescribed androgens, screening cardiometabolic risk, treating infection, referring high-risk patients early, and involving male partners in fertility conversations.

### 9.5 Assisted reproductive and fertility support services

Assisted reproductive technologies can help when prevention and treatment do not restore natural conception within a suitable timeframe. Options include intrauterine insemination, in vitro fertilisation, intracytoplasmic sperm injection, surgical sperm retrieval, donor sperm, and counselling about other family-building routes, chosen according to cause, prognosis, partner factors, cost, and patient values (1, 8, 9b).

Access to assisted reproduction should be paired with realistic counselling about success rates, multiple gestation, treatment burden, legal and ethical requirements, and the availability of cryopreserved sperm. Financial barriers and stigma can influence uptake, making equitable service design a public-health issue as well as a clinical one (1, 13).

Psychological support should accompany treatment because procedures can be expensive, emotionally demanding, and socially isolating. Respectful couple-centred counselling can reduce blame, improve adherence, and support informed choices even when biological parenthood is not immediately achievable (1, 2).

## 10 Public health, policy, and research implications

### 10.1 Public health implications

Male reproductive health should be integrated into public-health programmes on non-communicable disease, occupational safety, environmental protection, sexual health, mental health, substance-use care, and cancer survivorship (1, 12). Public communication should avoid stigma and deterministic claims while making evidence-based assessment and prevention visible to men and couples (13, 15).

### 10.2 Clinical implications

Clinicians need an integrated model of care. A reproductive history belongs in oncology, endocrinology, urology, primary care, nephrology, cardiology, psychiatry, occupational medicine, and sexual-health practice (2, 96). Men with abnormal semen results need structured history, examination, repeat testing, medication review, endocrine evaluation when indicated, and referral based on severity and the couple’s timeline (1, 9).

### 10.3 Policy implications

Policy should prioritise prevention at source: enforce occupational limits, strengthen pesticide controls, protect informal workers, improve air and water quality, control industrial emissions and e-waste, and regulate hazardous chemicals transparently (1, 10). Health systems should also embed fertility preservation in cancer pathways and reduce barriers to semen, endocrine, genetic, mental-health, and assisted-reproduction services (13, 59).

### 10.4 Research gaps

Several knowledge gaps remain. Longitudinal preconception cohorts with repeated exposure measures are needed to distinguish short-lived biomarker variation from sustained reproductive risk, to evaluate chemical and non-chemical mixtures, and to link semen or molecular markers with time-to-pregnancy, miscarriage, and live-birth outcomes. Better evidence is also needed on medication-specific effects, disease-treatment interactions, recovery after anabolic-androgenic steroid use, reproductive effects of contemporary antiretroviral regimens, occupational and climate-related heat, and the clinical validity of emerging epigenetic, microRNA, extracellular-vesicle, metabolomic, and proteomic biomarkers (16, 18, 29, 102).

Protocol registration, open reporting of null results, and standardised exposure definitions would further reduce publication bias and improve cross-regional evidence synthesis (16, 18).

Low- and middle-income settings require particular attention because informal work, pesticide use, contaminated water, air pollution, e-waste, limited laboratory capacity, and barriers to specialist care may coexist. More context-specific research can prevent the inappropriate transfer of risk estimates and clinical pathways from high-income populations without adaptation (13, 14).

## 11 Conclusion

Male reproductive health is shaped by the interaction of environmental exposures, pharmacological agents, substance use, chronic and infectious diseases, lifestyle practices, and broader social conditions. Environmental toxicants such as pesticides, heavy metals, endocrine-disrupting chemicals, air pollutants, and occupational heat may impair spermatogenesis, hormonal balance, sperm motility, sperm DNA integrity, and sexual function. Similarly, selected medications and substances, particularly chemotherapy, radiotherapy, exogenous testosterone, anabolic steroids, opioids, psychotropic drugs, and excessive alcohol use, may affect fertility through direct gonadal injury, endocrine suppression, vascular dysfunction, or altered sexual performance. Disease conditions including diabetes, obesity, varicocele, sexually transmitted infections, cardiovascular disorders, renal and liver disease, genetic abnormalities, neurological disorders, cancer, and psychological illness may further compromise fertility through oxidative stress, inflammation, hormonal disturbance, reproductive-tract obstruction, erectile dysfunction, and impaired ejaculation. Therefore, male infertility should not be interpreted as a single-condition problem but as a multifactorial reproductive-health concern requiring comprehensive assessment.

This review supports integration of male reproductive-health services into primary care, occupational health, oncology, endocrinology, urology, cardiology, nephrology, psychiatry, and sexual-health programmes. Health professionals should routinely assess occupational exposure, medication history, substance use, chronic illness, sexual function, reproductive goals, and psychosocial wellbeing when managing men with fertility concerns. Early semen analysis, targeted hormonal testing, appropriate genetic evaluation, infection screening, and specialist referral should be used where clinically indicated. Men exposed to gonadotoxic cancer treatments should receive timely fertility-preservation counselling before treatment begins. Governments, employers, and public-health institutions should strengthen pesticide regulation, workplace heat protection, air-pollution control, hazardous-waste management, and access to reproductive and mental-health services. Future research should prioritise long-term effects of combined environmental exposures, medication-specific reproductive outcomes, recovery after anabolic-androgenic steroid use, contemporary antiretroviral regimens, and fertility patterns in low- and middle-income settings.
