# Supplementary material for: Male reproductive health under environmental, pharmacological, and disease influences: a scoping review
Source: Front Endocrinol (Lausanne). 2026 Oct 2;17:1922443. doi: 10.3389/fendo.2026.1922443 (PMC13673332; doi:10.3389/fendo.2026.1922443)
Supplement: Supplementary file 1 [file Table1.docx]

**Supplementary Materials**

**Male Reproductive Health under Environmental, Pharmacological, and Disease Influences: A Scoping Review**

The evidence map comprises 105 sources: 99 PubMed-indexed peer-reviewed sources and six official grey-literature/guideline sources. Google Scholar was supplementary for discovery and citation chaining. Four methodological references used to guide scoping-review conduct and reporting are not included in the evidence-map count.

**Supplementary Table S1. Reproducible PubMed and Google Scholar search strategy used for the scoping review**

| **Source/domain** | **Exact search logic / approach** |
| --- | --- |
| PubMed – environmental domain | ("male infertility"[Title/Abstract] OR "male reproductive health"[Title/Abstract] OR semen[Title/Abstract] OR sperm[Title/Abstract] OR spermatogenesis[Title/Abstract]) AND (environment*[Title/Abstract] OR pollut*[Title/Abstract] OR pesticide*[Title/Abstract] OR "endocrine disrupt*"[Title/Abstract] OR phthalate*[Title/Abstract] OR bisphenol*[Title/Abstract] OR PFAS[Title/Abstract] OR "heavy metal*"[Title/Abstract] OR "air pollution"[Title/Abstract] OR heat[Title/Abstract] OR climate[Title/Abstract] OR radiation[Title/Abstract]) |
| PubMed – pharmacological domain | ("male infertility"[Title/Abstract] OR "male reproductive health"[Title/Abstract] OR semen[Title/Abstract] OR spermatogenesis[Title/Abstract]) AND (drug*[Title/Abstract] OR medication*[Title/Abstract] OR pharmacolog*[Title/Abstract] OR chemotherapy[Title/Abstract] OR radiotherapy[Title/Abstract] OR testosterone[Title/Abstract] OR "anabolic steroid*"[Title/Abstract] OR opioid*[Title/Abstract] OR antidepressant*[Title/Abstract] OR antipsychotic*[Title/Abstract] OR antihypertensive*[Title/Abstract] OR antiretroviral*[Title/Abstract] OR immunosuppress*[Title/Abstract]) |
| PubMed– disease domain | ("male infertility"[Title/Abstract] OR "male reproductive health"[Title/Abstract] OR semen[Title/Abstract] OR spermatogenesis[Title/Abstract]) AND (diabetes[Title/Abstract] OR obesity[Title/Abstract] OR "metabolic syndrome"[Title/Abstract] OR varicocele[Title/Abstract] OR infection*[Title/Abstract] OR HIV[Title/Abstract] OR cardiovascular[Title/Abstract] OR kidney[Title/Abstract] OR renal[Title/Abstract] OR liver[Title/Abstract] OR genetic*[Title/Abstract] OR neurolog*[Title/Abstract] OR cancer[Title/Abstract] OR autoimmune[Title/Abstract]) |
| PubMed – mechanisms, mixtures and biomarkers | (sperm[Title/Abstract] OR semen[Title/Abstract] OR "male infertility"[Title/Abstract]) AND ("oxidative stress"[Title/Abstract] OR inflammation[Title/Abstract] OR "endocrine disruption"[Title/Abstract] OR "DNA fragmentation"[Title/Abstract] OR epigenetic*[Title/Abstract] OR "DNA methylation"[Title/Abstract] OR "histone retention"[Title/Abstract] OR microRNA[Title/Abstract] OR "small RNA"[Title/Abstract] OR "extracellular vesicle*"[Title/Abstract] OR proteomic*[Title/Abstract] OR mixture*[Title/Abstract] OR "weighted quantile sum"[Title/Abstract] OR BKMR[Title/Abstract] OR "quantile g-computation"[Title/Abstract]) |

**Supplementary Table S2. Complete source-level evidence map using APA author–date citations, with quantitative findings reported where available**

Source classification: PubMed-indexed peer-reviewed evidence sources, n=99; official grey literature/guidelines, n=6; total mapped evidence sources, n=105. Google Scholar was supplementary for discovery/citation chaining and did not contribute a unique Google-Scholar-only journal record after de-duplication.

|  | **Domain** | **reference** | **Design/sample** | **Quantitative/evidential contribution** |
| --- | --- | --- | --- | --- |
| 1. | Cross-domain/context | Abarikwu (2013) | Review/primary study/guideline | Qualitative contribution to the mapped domain; no single pooled numerical estimate was required for the way this source was used. |
| 2. | Population burden/context | Agarwal et al. (2015) | Review/primary study/guideline | Qualitative contribution to the mapped domain; no single pooled numerical estimate was required for the way this source was used. |
| 3. | Environmental/exposure | Akhatova et al. (2025) | Review/primary study/guideline | Qualitative contribution to the mapped domain; no single pooled numerical estimate was required for the way this source was used. |
| 4. | Disease/clinical | Akhigbe et al. (2022) | Review/primary study/guideline | Qualitative contribution to the mapped domain; no single pooled numerical estimate was required for the way this source was used. |
| 5. | Environmental/exposure | Akhigbe et al. (2024) | Systematic review/meta-analysis; 12 studies, 2,050 men | Overall sperm motility was lower; count and concentration effects varied by region and heterogeneity was substantial. |
| 6. | Disease/clinical | Andreoli et al. (2023) | Review/primary study/guideline | Qualitative contribution to the mapped domain; no single pooled numerical estimate was required for the way this source was used. |
| 7. | Pharmacological/treatment | Asghar et al. (2024) | Meta-analysis; 7 preclinical studies (112 animals) and 5 clinical studies (274 men) | In clinical studies, valproate was associated with lower sperm motility (SMD −1.62, 95% CI −2.81 to −0.43); sperm count, abnormal morphology, FSH, and LH differences were not statistically significant. |
| 8. | Lifestyle/behaviour | Belladelli et al. (2023) | Review/primary study/guideline | Qualitative contribution to the mapped domain; no single pooled numerical estimate was required for the way this source was used. |
| 9. | Environmental/exposure | Bloom et al. (2015) | Review/primary study/guideline | Qualitative contribution to the mapped domain; no single pooled numerical estimate was required for the way this source was used. |
| 10. | Mechanisms/methods/guidance | Boitrelle et al. (2021) | Review/primary study/guideline | Qualitative contribution to the mapped domain; no single pooled numerical estimate was required for the way this source was used. |
| 11. | Pharmacological/treatment | Bradley & Boland (2023) | Review/primary study/guideline | Qualitative contribution to the mapped domain; no single pooled numerical estimate was required for the way this source was used. |
| 12. | Mechanisms/methods/guidance | Brannigan et al. (2024) | Review/primary study/guideline | Qualitative contribution to the mapped domain; no single pooled numerical estimate was required for the way this source was used. |
| 13. | Lifestyle/behaviour | Bundhun et al. (2019) | Review/primary study/guideline | Qualitative contribution to the mapped domain; no single pooled numerical estimate was required for the way this source was used. |
| 14. | Disease/clinical | Campbell et al. (2026) | Systematic review/meta-analysis; 51 studies qualitative, 35 quantitative | HPV was associated with antisperm antibodies (OR 10.63, 95% CI 1.49–75.93); HBV with miscarriage (OR 1.43, 1.05–1.93); many pregnancy/live-birth outcomes were nonsignificant. |
| 15. | Cross-domain/context | Cannarella et al. (2019) | Review/primary study/guideline | Qualitative contribution to the mapped domain; no single pooled numerical estimate was required for the way this source was used. |
| 16. | Disease/clinical | Cannarella et al. (2024) | Meta-analysis; 351 studies (23 RCTs, 292 observational, 36 case-control) | After varicocele repair: concentration SMD 1.590, total count 1.824, total motile count 1.643, progressive motility 1.845, total motility 1.613, and morphology 1.066; heterogeneity was very high for most outcomes. |
| 17. | Environmental/exposure | Checa Vizcaíno et al. (2016) | Review/primary study/guideline | Qualitative contribution to the mapped domain; no single pooled numerical estimate was required for the way this source was used. |
| 18. | Mechanisms/methods/guidance | Chung et al. (2022) | Review/primary study/guideline | Qualitative contribution to the mapped domain; no single pooled numerical estimate was required for the way this source was used. |
| 19. | Mechanisms/methods/guidance | Cocuzza & Esteves (2014) | Review/primary study/guideline | Qualitative contribution to the mapped domain; no single pooled numerical estimate was required for the way this source was used. |
| 20. | Pharmacological/treatment | Desai et al. (2022) | Review/primary study/guideline | Qualitative contribution to the mapped domain; no single pooled numerical estimate was required for the way this source was used. |
| 21. | Pharmacological/treatment | Esposito et al. (2023) | Review/primary study/guideline | Qualitative contribution to the mapped domain; no single pooled numerical estimate was required for the way this source was used. |
| 22. | Mechanisms/methods/guidance | Esteves et al. (2021) | Evidence synthesis/practice recommendations | Forty-one recommendations were formulated: 13 technical/testing recommendations and 28 indication/management recommendations; underlying evidence was largely moderate-to-low quality. |
| 23. | Mechanisms/methods/guidance | European Association of Urology. (2026) | Review/primary study/guideline | Qualitative contribution to the mapped domain; no single pooled numerical estimate was required for the way this source was used. |
| 24. | Disease/clinical | Facondo et al. (2022) | Systematic review/meta-analysis; 14 studies, 8 semen studies meta-analysed | DM1 was associated with lower normal morphology (MD −0.36 percentage points) and progressive motility (MD −33.62 percentage points), with no clear difference in total count/concentration. |
| 25. | Disease/clinical | Garolla et al. (2024) | Review/primary study/guideline | Qualitative contribution to the mapped domain; no single pooled numerical estimate was required for the way this source was used. |
| 26. | Environmental/exposure | Giulioni et al. (2022) | Systematic review; 64 human studies | Recurring adverse findings for semen parameters and sperm DNA/chromatin integrity; substantial heterogeneity across pesticide classes and designs. |
| 27. | Environmental/exposure | Hamed et al. (2023) | Systematic review/meta-analysis; 766 men (349 exposed, 417 controls) | Organophosphate exposure was associated with lower sperm count, concentration, total and progressive motility, and normal morphology; count and progressive-motility effects were not robust in all sensitivity analyses, and testosterone did not differ significantly. |
| 28. | Environmental/exposure | Hoang-Thi et al. (2022) | Meta-analysis; 9 studies, 356 men | High-temperature exposure was associated with lower sperm concentration (SMD −1.07) and motility (SMD −1.93), with several other semen parameters also reduced. |
| 29. | Environmental/exposure | Huang et al. (2020) | Review/primary study/guideline | Qualitative contribution to the mapped domain; no single pooled numerical estimate was required for the way this source was used. |
| 30. | Pharmacological/treatment | Huijben et al. (2023) | Systematic review/meta-analysis; 18 studies, 731 men; 15 studies/566 in meta-analysis | Clomiphene increased sperm concentration by 8.38×10^6/mL and total motility by 8.14 percentage points; mean pregnancy rate across 10 studies was 17%. |
| 31. | Lifestyle/behaviour | Ilacqua et al. (2018) | Review/primary study/guideline | Qualitative contribution to the mapped domain; no single pooled numerical estimate was required for the way this source was used. |
| 32. | Disease/clinical | James et al. (2025) | Review/primary study/guideline | Qualitative contribution to the mapped domain; no single pooled numerical estimate was required for the way this source was used. |
| 33. | Environmental/exposure | Ji et al. (2018) | Review/primary study/guideline | Qualitative contribution to the mapped domain; no single pooled numerical estimate was required for the way this source was used. |
| 34. | Pharmacological/treatment | Junqueira et al. (2023) | Review/primary study/guideline | Qualitative contribution to the mapped domain; no single pooled numerical estimate was required for the way this source was used. |
| 35. | Environmental/exposure | Jurewicz et al. (2018) | Review/primary study/guideline | Qualitative contribution to the mapped domain; no single pooled numerical estimate was required for the way this source was used. |
| 36. | Environmental/exposure | Kakavandi et al. (2023) | Review/primary study/guideline | Qualitative contribution to the mapped domain; no single pooled numerical estimate was required for the way this source was used. |
| 37. | Environmental/exposure | Kaur et al. (2024) | Systematic review/meta-analysis | Blood lead SMD 0.67 (95% CI 0.07–1.28), seminal lead SMD 1.19 (0.42–1.96), and seminal cadmium SMD 0.92 (0.54–1.29). |
| 38. | Environmental/exposure | Knapke et al. (2022) | Review/primary study/guideline | Qualitative contribution to the mapped domain; no single pooled numerical estimate was required for the way this source was used. |
| 39. | Disease/clinical | Korchia et al. (2023) | Review/primary study/guideline | Qualitative contribution to the mapped domain; no single pooled numerical estimate was required for the way this source was used. |
| 40. | Disease/clinical | Krausz & Casamonti (2017) | Review/primary study/guideline | Qualitative contribution to the mapped domain; no single pooled numerical estimate was required for the way this source was used. |
| 41. | Disease/clinical | Krausz & Riera-Escamilla (2018) | Review/primary study/guideline | Qualitative contribution to the mapped domain; no single pooled numerical estimate was required for the way this source was used. |
| 42. | Population burden/context | Kumar & Singh (2015) | Review/primary study/guideline | Qualitative contribution to the mapped domain; no single pooled numerical estimate was required for the way this source was used. |
| 43. | Disease/clinical | Köhler et al. (2024) | Review/primary study/guideline | Qualitative contribution to the mapped domain; no single pooled numerical estimate was required for the way this source was used. |
| 44. | Disease/clinical | Leggio et al. (2025) | Review/primary study/guideline | Qualitative contribution to the mapped domain; no single pooled numerical estimate was required for the way this source was used. |
| 45. | Mechanisms/methods/guidance | Levine et al. (2023) | Systematic review/meta-regression | Mean sperm concentration declined 51.6% across samples collected from 1973 to 2018; this is a population temporal signal, not proof of a specific exposure. |
| 46. | Disease/clinical | Li et al. (2024) | Systematic review/meta-analysis; 50 studies, 71,337 participants | Obesity was associated with lower semen volume, total sperm number, total/progressive motility, and normal morphology. |
| 47. | Cross-domain/context | Liu et al. (2018) | Review/primary study/guideline | Qualitative contribution to the mapped domain; no single pooled numerical estimate was required for the way this source was used. |
| 48. | Pharmacological/treatment | Lou et al. (2023) | Review/primary study/guideline | Qualitative contribution to the mapped domain; no single pooled numerical estimate was required for the way this source was used. |
| 49. | Disease/clinical | Lundy & Vij (2019) | Review/primary study/guideline | Qualitative contribution to the mapped domain; no single pooled numerical estimate was required for the way this source was used. |
| 50. | Environmental/exposure | Lü et al. (2024) | Systematic review/meta-analysis; 18 studies | Urinary BPA was inversely associated with sperm concentration (β=−0.03, 95% CI −0.06 to −0.01) and total sperm count (β=−0.05, −0.08 to −0.02). |
| 51. | Disease/clinical | Malini et al. (2026) | Review/primary study/guideline | Qualitative contribution to the mapped domain; no single pooled numerical estimate was required for the way this source was used. |
| 52. | Cross-domain/context | Mann et al. (2020) | Review/primary study/guideline | Qualitative contribution to the mapped domain; no single pooled numerical estimate was required for the way this source was used. |
| 53. | Environmental/exposure | Margiana et al. (2026) | Review/primary study/guideline | Qualitative contribution to the mapped domain; no single pooled numerical estimate was required for the way this source was used. |
| 54. | Environmental/exposure | Marić et al. (2021) | Review/primary study/guideline | Qualitative contribution to the mapped domain; no single pooled numerical estimate was required for the way this source was used. |
| 55. | Environmental/exposure | Martenies & Perry (2013) | Review/primary study/guideline | Qualitative contribution to the mapped domain; no single pooled numerical estimate was required for the way this source was used. |
| 56. | Environmental/exposure | Martínez et al. (2023) | Review/primary study/guideline | Qualitative contribution to the mapped domain; no single pooled numerical estimate was required for the way this source was used. |
| 57. | Pharmacological/treatment | McBride & Coward (2016) | Review/primary study/guideline | Qualitative contribution to the mapped domain; no single pooled numerical estimate was required for the way this source was used. |
| 58. | Environmental/exposure | Mehrpour et al. (2014) | Review/primary study/guideline | Qualitative contribution to the mapped domain; no single pooled numerical estimate was required for the way this source was used. |
| 59. | Pharmacological/treatment | Melhem et al. (2026) | Systematic review/meta-analysis; 41 RCTs, 3,615 men | Antioxidants: sperm concentration SMD 1.09, progressive motility 1.11, total motility 0.69, morphology 0.43; certainty was very low and prediction intervals crossed the null. |
| 60. | Cross-domain/context | Milosavljević et al. (2022) | Review/primary study/guideline | Qualitative contribution to the mapped domain; no single pooled numerical estimate was required for the way this source was used. |
| 61. | Environmental/exposure | Mima et al. (2018) | Review/primary study/guideline | Qualitative contribution to the mapped domain; no single pooled numerical estimate was required for the way this source was used. |
| 62. | Mechanisms/methods/guidance | Minhas et al. (2025) | Review/primary study/guideline | Qualitative contribution to the mapped domain; no single pooled numerical estimate was required for the way this source was used. |
| 63. | Environmental/exposure | Mizuno et al. (2025) | Cross-sectional study; 197 men | WQS, q-g-computation, and BKMR consistently identified mixture associations with motility and reproductive hormones; no significant association with semen volume, count, or concentration. |
| 64. | Pharmacological/treatment | Mouyis et al. (2019) | Review/primary study/guideline | Qualitative contribution to the mapped domain; no single pooled numerical estimate was required for the way this source was used. |
| 65. | Cross-domain/context | Mínguez-Alarcón et al. (2023) | Review/primary study/guideline | Qualitative contribution to the mapped domain; no single pooled numerical estimate was required for the way this source was used. |
| 66. | Mechanisms/methods/guidance | National Institute for Health and Care Excellence. (2026) | Review/primary study/guideline | Qualitative contribution to the mapped domain; no single pooled numerical estimate was required for the way this source was used. |
| 67. | Mechanisms/methods/guidance | Oehninger & Ombelet (2019) | Review/primary study/guideline | Qualitative contribution to the mapped domain; no single pooled numerical estimate was required for the way this source was used. |
| 68. | Environmental/exposure | Omolaoye et al. (2022) | Review/primary study/guideline | Qualitative contribution to the mapped domain; no single pooled numerical estimate was required for the way this source was used. |
| 69. | Disease/clinical | Peel et al. (2026) | Review/primary study/guideline | Qualitative contribution to the mapped domain; no single pooled numerical estimate was required for the way this source was used. |
| 70. | Environmental/exposure | Pereira et al. (2020) | Review/primary study/guideline | Qualitative contribution to the mapped domain; no single pooled numerical estimate was required for the way this source was used. |
| 71. | Environmental/exposure | Perry (2008) | Review/primary study/guideline | Qualitative contribution to the mapped domain; no single pooled numerical estimate was required for the way this source was used. |
| 72. | Environmental/exposure | Pizzol et al. (2021) | Review/primary study/guideline | Qualitative contribution to the mapped domain; no single pooled numerical estimate was required for the way this source was used. |
| 73. | Environmental/exposure | Presunto et al. (2023) | Review/primary study/guideline | Qualitative contribution to the mapped domain; no single pooled numerical estimate was required for the way this source was used. |
| 74. | Cross-domain/context | Rahban & Nef (2020) | Review/primary study/guideline | Qualitative contribution to the mapped domain; no single pooled numerical estimate was required for the way this source was used. |
| 75. | Pharmacological/treatment | Rahnema et al. (2014) | Review/primary study/guideline | Qualitative contribution to the mapped domain; no single pooled numerical estimate was required for the way this source was used. |
| 76. | Environmental/exposure | Ramsay et al. (2023) | Retrospective cohort; 21,563 men | Residential exposure to industrial air emissions was associated with poorer semen/fertility outcomes; exposure modelling and observational design limit causal inference. |
| 77. | Environmental/exposure | Rodprasert et al. (2023) | Review/primary study/guideline | Qualitative contribution to the mapped domain; no single pooled numerical estimate was required for the way this source was used. |
| 78. | Lifestyle/behaviour | Rotimi & Singh (2024) | Review/primary study/guideline | Qualitative contribution to the mapped domain; no single pooled numerical estimate was required for the way this source was used. |
| 79. | Disease/clinical | Salas-Huetos et al. (2021) | Systematic review; 60 qualitative and 28 quantitative studies | Overweight/obesity was associated with lower semen volume, sperm count/concentration, vitality, total motility, normal morphology, and adverse reproductive-hormone profiles. |
| 80. | Cross-domain/context | Samplaski & Nangia (2015) | Review/primary study/guideline | Qualitative contribution to the mapped domain; no single pooled numerical estimate was required for the way this source was used. |
| 81. | Cross-domain/context | Santi et al. (2025) | Meta-analysis; 12 studies, 345 men | Weight-loss interventions were associated with improvements in sperm concentration, progressive motility, and DNA fragmentation. |
| 82. | Mechanisms/methods/guidance | Schlegel et al. (2021a) | Review/primary study/guideline | Qualitative contribution to the mapped domain; no single pooled numerical estimate was required for the way this source was used. |
| 83. | Mechanisms/methods/guidance | Schlegel et al. (2021a) | Review/primary study/guideline | Qualitative contribution to the mapped domain; no single pooled numerical estimate was required for the way this source was used. |
| 84. | Environmental/exposure | Sciorio et al. (2025) | Review/primary study/guideline | Qualitative contribution to the mapped domain; no single pooled numerical estimate was required for the way this source was used. |
| 85. | Pharmacological/treatment | Semet et al. (2017) | Review/primary study/guideline | Qualitative contribution to the mapped domain; no single pooled numerical estimate was required for the way this source was used. |
| 86. | Population trend/context | Sengupta et al. (2017) | Meta-analysis; 14 African studies | Reported a 72.6% decrease in mean sperm concentration from 1965 to 2015; historical heterogeneity requires cautious interpretation. |
| 87. | Pharmacological/treatment | Shahid et al. (2021) | Review/primary study/guideline | Qualitative contribution to the mapped domain; no single pooled numerical estimate was required for the way this source was used. |
| 88. | Cross-domain/context | Sharma et al. (2020) | Review/primary study/guideline | Qualitative contribution to the mapped domain; no single pooled numerical estimate was required for the way this source was used. |
| 89. | Lifestyle/behaviour | Sharma et al. (2013) | Review/primary study/guideline | Qualitative contribution to the mapped domain; no single pooled numerical estimate was required for the way this source was used. |
| 90. | Lifestyle/behaviour | Sharma et al. (2016) | Meta-analysis; 20 studies, 5,865 men | Smoking was associated with lower sperm count (MD −9.72×10^6/mL), motility (−3.48 percentage points), and morphology (−1.37 percentage points). |
| 91. | Environmental/exposure | Skakkebaek et al. (2016) | Review/primary study/guideline | Qualitative contribution to the mapped domain; no single pooled numerical estimate was required for the way this source was used. |
| 92. | Disease/clinical | Smith et al. (2025) | Review/primary study/guideline | Qualitative contribution to the mapped domain; no single pooled numerical estimate was required for the way this source was used. |
| 93. | Pharmacological/treatment | Su et al. (2025) | Review/primary study/guideline | Qualitative contribution to the mapped domain; no single pooled numerical estimate was required for the way this source was used. |
| 94. | Environmental/exposure | Szumilas et al. (2020) | Review/primary study/guideline | Qualitative contribution to the mapped domain; no single pooled numerical estimate was required for the way this source was used. |
| 95. | Environmental/exposure | Tesarik (2025) | Review/primary study/guideline | Qualitative contribution to the mapped domain; no single pooled numerical estimate was required for the way this source was used. |
| 96. | Mechanisms/methods/guidance | Vitthala et al. (2026) | Systematic review/meta-analysis; 52 studies, 19,930 subjects | High SDF showed low sensitivity, higher specificity, poor-to-moderate discrimination, assay-dependent cut-offs, and substantial heterogeneity. |
| 97. | Pharmacological/treatment | Wang et al. (2025) | Network meta-analysis; 30 RCTs, 18,157 participants | Compared with placebo, ejaculation-dysfunction risk was higher with clomipramine (OR 42.11), WS5570 (OR 28.99), and paroxetine (OR 18.63); erectile-dysfunction risk was also higher with several agents, including duloxetine and escitalopram. |
| 98. | Disease/clinical | Wang et al. (2020) | Meta-analysis; 4 RCTs, 349 participants | No statistically clear improvement in spontaneous pregnancy after primary varicocele surgery. |
| 99. | Mechanisms/methods/guidance | World Health Organization. (2023a) | Review/primary study/guideline | Qualitative contribution to the mapped domain; no single pooled numerical estimate was required for the way this source was used. |
| 100. | Cross-domain/context | World Health Organization. (2023b). Infertility. ht | Review/primary study/guideline | Qualitative contribution to the mapped domain; no single pooled numerical estimate was required for the way this source was used. |
| 101. | Mechanisms/methods/guidance | World Health Organization. (2025) | Review/primary study/guideline | Qualitative contribution to the mapped domain; no single pooled numerical estimate was required for the way this source was used. |
| 102. | Mechanisms/methods/guidance | World Health Organization. (2021) | Review/primary study/guideline | Qualitative contribution to the mapped domain; no single pooled numerical estimate was required for the way this source was used. |
| 103. | Environmental/exposure | Xu et al. (2023) | Systematic review/meta-analysis; 11 studies | Per 10 μg/m³ increase: PM10 was associated with 2.18% lower sperm concentration and 2.76% lower total sperm number; SO2 with 8.61% lower concentration and 9.52% lower total number; PM2.5 and PM10 were associated with lower total and progressive motility. |
| 104. | Environmental/exposure | Zhao et al. (2022) | Cohort; 33,876 men from 340 Chinese cities | PM2.5/PM10 exposure was associated with lower total/progressive motility and greater asthenozoospermia risk. |
| 105. | Disease/clinical | Ziouziou et al. (2026) | Systematic review/meta-analysis; 32 studies from 1,622 records | Varicocele was associated with higher FSH (MD 2.18 IU/L), higher LH (0.63 IU/L), and lower total testosterone (−0.71 ng/mL). |
